# Human yolk sac-like haematopoiesis generates *RUNX1-*, *GFI1-* and/or *GFI1B-*dependent blood and *SOX17*-positive endothelium

**DOI:** 10.1242/dev.193037

**Published:** 2020-10-29

**Authors:** Freya F. Bruveris, Elizabeth S. Ng, Ana Rita Leitoguinho, Ali Motazedian, Katerina Vlahos, Koula Sourris, Robyn Mayberry, Penelope McDonald, Lisa Azzola, Nadia M. Davidson, Alicia Oshlack, Edouard G. Stanley, Andrew G. Elefanty

**Affiliations:** 1 Murdoch Children's Research Institute, The Royal Children's Hospital, Flemington Road, Parkville, Victoria 3052, Australia; 2 Department of Paediatrics, Faculty of Medicine, Dentistry and Health Sciences, University of Melbourne, Parkville, Victoria 3052, Australia; 3 School of BioSciences, University of Melbourne, Parkville, Victoria 3052, Australia; 4 Department of Anatomy and Developmental Biology, Monash University, Clayton, Victoria 3800, Australia

**Keywords:** Yolk sac haematopoiesis, Human pluripotent stem cells, RUNX1, GFI1/1B

## Abstract

The genetic regulatory network controlling early fate choices during human blood cell development are not well understood. We used human pluripotent stem cell reporter lines to track the development of endothelial and haematopoietic populations in an *in vitro* model of human yolk-sac development. We identified SOX17^−^CD34^+^CD43^−^ endothelial cells at day 2 of blast colony development, as a haemangioblast-like branch point from which SOX17^−^CD34^+^CD43^+^ blood cells and SOX17^+^CD34^+^CD43^−^ endothelium subsequently arose. Most human blood cell development was dependent on RUNX1. Deletion of *RUNX1* only permitted a single wave of yolk sac-like primitive erythropoiesis, but no yolk sac myelopoiesis or aorta-gonad-mesonephros (AGM)-like haematopoiesis. Blocking GFI1 and/or GFI1B activity with a small molecule inhibitor abrogated all blood cell development, even in cell lines with an intact *RUNX1* gene. Together, our data define the hierarchical requirements for RUNX1, GFI1 and/or GFI1B during early human haematopoiesis arising from a yolk sac-like SOX17-negative haemogenic endothelial intermediate.

## Abstract (precis)

**Highlighted Article:** The hierarchical requirements for RUNX1, GFI1 and/or GFI1B during early human haematopoiesis arising from a yolk sac-like haemogenic endothelial intermediate.

## INTRODUCTION

Blood cells develop from an endothelial intermediate at multiple stages during embryonic development in vertebrates (Dzierzak and Bigas, 2018; Ivanovs et al., 2017). Studies in the mouse have revealed that this is true for erythro-myeloid progenitors (EMPs) arising from yolk sac endothelium, and for blood cells emerging from aortic endothelium in the aorta-gonad-mesonephros (AGM) region (de Bruijn and Dzierzak, 2017; Frame et al., 2016; Okuda et al., 1996; Swiers et al., 2013; Wang et al., 1996). Similarities between mouse and human haematopoietic development (Dzierzak and Bigas, 2018; Ivanovs et al., 2017) suggest that the same regulatory genes crucial for mouse haematopoietic development will play essential roles in blood formation in the human embryo. As studies of genetically modified human blood cells in the context of a developing human embryo are not possible, haematopoietic differentiation of human pluripotent stem cells has emerged as the most tractable surrogate experimental system.

The methylcellulose-based blast colony-forming cell (BL-CFC) assay has been used to interrogate formation of the earliest human blood cells that correspond to the first products of yolk sac haematopoiesis (Kennedy et al., 2007). In this assay, the BL-CFC proliferates and differentiates to form a core structure (D'Souza et al., 2005) that includes haemogenic endothelial cells, which in turn generate blood cells via an endothelial-to-haematopoietic transition (EHT) (Lancrin et al., 2009). Although haematopoiesis is orchestrated by numerous transcription factors, *RUNX1* is a fundamental regulator of this process (Gao et al., 2018; Thambyrajah et al., 2016b). EMPs from the mouse yolk sac, and HSCs and preHSCs emerging from the mouse AGM all require *Runx1*, whereas the earliest wave of yolk sac erythrocytes still appears in *Runx1*-null mouse embryos (de Bruijn and Dzierzak, 2017; Frame et al., 2016; Okuda et al., 1996; Wang et al., 1996).

Mindful of the ambiguity that surrounds the use of terms ‘primitive’ and ‘definitive’ to describe waves of haematopoiesis, here we have endeavoured to use nomenclature based on the embryonic site at which blood cells are made (Ivanovs et al., 2017). The term ‘extra-embryonic’ is applied to blood cells similar to those developing in the yolk sac, which do not express *HOXA* genes. This includes ‘primitive’ [erythroid, macrophage and megakaryocytic cells (McGrath et al., 2015a,b)] and ‘definitive’ [EMPs and yolk sac derived lymphoid cells (McGrath et al., 2015a; Yoshimoto et al., 2011, 2012)] waves of yolk sac haematopoiesis. ‘Intra-embryonic’ is applied to blood cells similar to those that develop in the AGM, that express *HOXA* genes in stem cells and progenitors, and that include the first repopulating HSCs, their precursors, and myeloid and lymphoid progeny (Ivanovs et al., 2017). This is also called ‘definitive’ haematopoiesis in the literature.

In this study, we have tracked the emergence of vascular and haematopoietic lineages using a human pluripotent stem cell (hPSC) line in which mCHERRY and GFP report expression of *SOX17* in endothelium and of the *RUNX1C* isoform of *RUNX1* in haematopoietic progenitors (Ng et al., 2016). By modelling extra-embryonic haematopoiesis in the blast colony assay, we show that differentiating *SOX17*^−^ endothelial-like cells acts like a haemangioblast population, because they give rise to the majority of blood cells and also to a *SOX17*^+^ endothelium. Blood formation was *RUNX1* dependent, because deletion of *RUNX1* resulted in the failure of normal blast colony development, with replacement of mixed haematopoietic and vascular colonies by reduced numbers of core structures containing *SOX17*^+^ endothelia but no blood cells. A single wave of extra-embryonic erythropoiesis, without macrophage formation, was possible in the absence of *RUNX1*, but was only revealed under specific culture conditions on an air-liquid interface. Furthermore, this wave of *RUNX1*-independent erythropoiesis was blocked by a small molecule inhibitor of *GFI1* and/or *GFI1B* (*GFI1/1B*) signalling. As expected, *RUNX1*-deficient hPSCs were unable to form blood cells upon differentiation towards *HOXA*^+^ intra-embryonic haematopoiesis. In summary, we show that human blood cell development from differentiating hPSCs under extra embryonic, yolk sac-like conditions arises from a haemangioblast-like *SOX17*^−^ endothelial cell, and is sequentially dependent upon *GFI1/1B* and *RUNX1*.

## RESULTS

To facilitate the dissection of early human haematopoiesis, we used a dual reporter hPSC line, *SOX17*^mCHERRY/w^*RUNX1C*^GFP/w^ (hereafter denoted SOX-RUNX) (Ng et al., 2016), in which GFP targeted to *RUNX1C* marks hematopoietic progenitor cells (Corada et al., 2013; Sroczynska et al., 2009), and mCHERRY targeted to *SOX17* marks vascular endothelium (Burtscher et al., 2012; Challen and Goodell, 2010; Clarke et al., 2013).

### Modelling extra-embryonic, yolk sac-like haematopoiesis

**Fig. 1.**
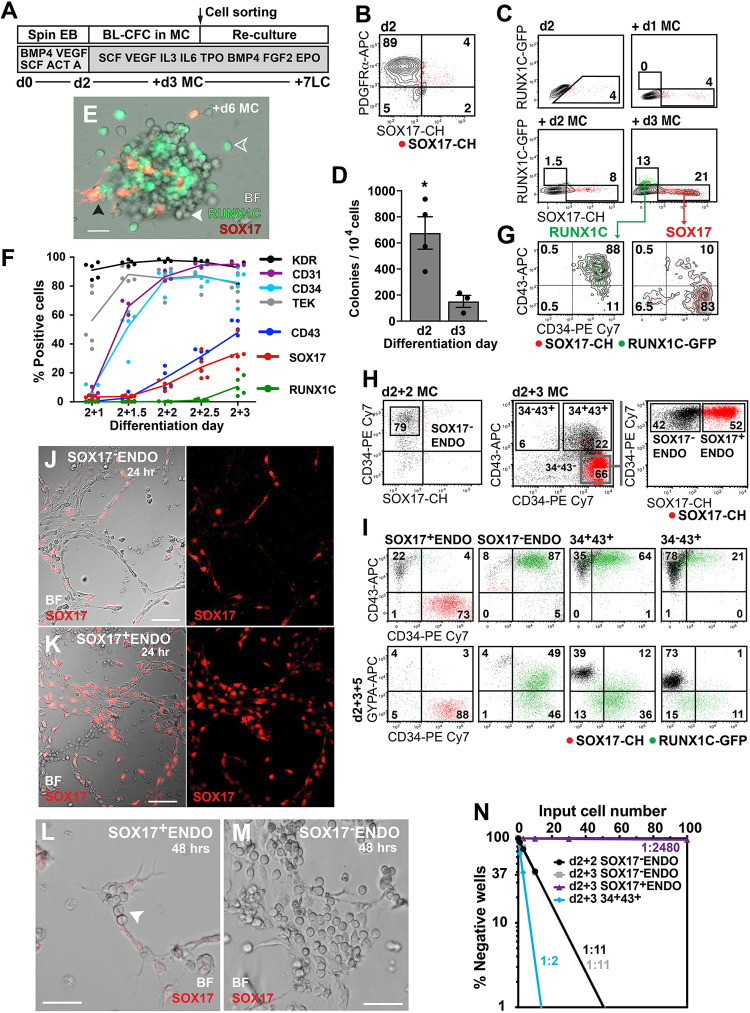
**SOX17-positive and SOX17-negative endothelium generate haematopoietic cells.** (A) Blast colony differentiation protocol. EB, embryoid body; BL-CFC, blast colony-forming cell; MC, methylcellulose; LC, liquid culture. (B) Flow-cytometry profile demonstrating that most d2 differentiated cells express PDGFRα (*n*=5 experiments). Data values are provided in the text. The data are shown as a contour plot to aid in the visualisation of the small numbers of events captured. This was also carried out in C and G. (C) Flow cytometric analysis of SOX17 and RUNX1C expression in d2 developing blast colonies following 1 (+d1) to 3 (+d3) days of methylcellulose (MC) culture (*n*=5 experiments). Data values are provided in the text. (D) Frequency of blast colonies was greater in d2 compared with d3 differentiated cells (mean±s.e.m., d2, *n*=4; d3, *n*=3 experiments). *P=0.018 (unpaired, Student's *t*-test). (E) Bright-field (BF) and merged fluorescent (RUNX1C and SOX17) image of a d6 blast colony with SOX17^+^ endothelial cells (black arrowhead), RUNX1C^+^ (hollow white arrowhead) and RUNX1C^−^ blood cells (solid white arrowhead). Scale bar: 100 µm. (F) Flow cytometry timecourse of differentiating blast colonies from d1 (2+1) to d3 (2+3) in MC culture showing expression of endothelial (KDR, CD31, CD34, TEK and SOX17) and haematopoietic (CD43 and RUNX1C) markers (*n*=3-5 experiments per marker). Data values are provided in the text. (G) At d3 of differentiation, RUNX1C identifies CD34^+^CD43^+^ blood cells and SOX17 marks CD34^+^ endothelial cells and some SOX17^+^CD34^+^CD43^+^ haematopoietic cells (*n*=5 experiments). Data values are provided in the text. (H) Sorting strategy to isolate endothelial (SOX17^+^ENDO, SOX17^−^ENDO) and haematopoietic (34^+^43^+^ or 34^+^43^−^) populations from d2 blast colonies at d2 (d2+2) and d3 (d2+3) of methylcellulose (MC) culture (*n*=8 experiments at d2+2; *n*=15 experiments at d2+3). (I) Analysis of sorted fractions showing CD34, CD43 and GYPA expression after 5 days of liquid culture (d2+3+5). Data for d2+3+3 and d2+3+7 time points is shown in Fig. S1B (*n*=3 experiments). (J-M) Images of d2+3 SOX17-sorted endothelial tube networks after 24 h (J,K) and 48 h (L,M) of culture, highlighting abundant blood cell formation from SOX17^−^ENDO compared with SOX17^+^ENDO sorted cells (white arrowhead). Scale bars: 50 µm in J,K; 100 µm in L,M. *n*=2 experiments. (N) Limit dilution analyses of d2+2 and d2+3 blast colony sorted fractions, correlating input cell number in each well with the frequency of wells in which haematopoietic clones were absent (% negative wells). The frequency of haemogenic and progenitor cells in each population is shown, calculated as the cell input number that gave 37% negative wells using Poisson statistics. Regression lines for d2+2 and d2+3 SOX17^−^ENDO overlie each other. Data represent the sum of three experiments. See Table S1.

SOX-RUNX cells were differentiated to haematopoietic mesoderm, dissociated and transferred into methylcellulose cultures for blast colony (BL-CFC) assays (Fig. 1A and Materials and Methods). Day 2 (d2) mesoderm cells expressed the mesendodermal marker PDGFRα (92.5±1.7%, *n*=5) (here and hereafter data represent mean±s.e.m. and the number of independent experiments) (Fig. 1B), from which BL-CFCs derive (Davis et al., 2008), but few cells expressed the SOX17-mCHERRY reporter (hereafter denoted SOX17) (7.2±1.4%, *n*=5) and RUNX1C-GFP-expressing cells (hereafter denoted RUNX1C) were not present (Fig. 1C). There was a fourfold higher frequency of BL-CFCs generated from d2 compared with d3 SOX-RUNX differentiated mesoderm cells (Fig. 1D).

Differentiated blast colonies comprised a mix of SOX17-positive and -negative adherent cells, and RUNX1C-positive and -negative haematopoietic cells (Fig. 1E). Tracking haematopoietic and endothelium development revealed that most cells expressed vascular endothelial growth factor receptor 2 (KDR) within the first day of methylcellulose culture, rapidly followed by expression of the haematovascular markers TEK (TIE2), CD31 and CD34 (Fig. 1F). SOX17 (11.4±3.1%, *n*=5) and CD43 (16.5±3.6%, *n*=5) expression appeared after 2 days of methylcellulose differentiation, delineating subsets of endothelial (SOX17^+^CD34^+^CD43^−^) and haematopoietic (CD43^+^) cells. RUNX1C expression (10.6±6.1%, *n*=5) was observed in one third (33.6±9.8%, *n*=5) of CD43^+^ cells (RUNX1C^+^CD34^+^CD43^+^) a day later, in a population largely excluding SOX17^+^ cells (Fig. 1C,F,G). As expected, most RUNX1C^+^ cells (76±13.3%, *n*=5) expressed CD43 (Fig. 1G). A small percentage of SOX17^+^ cells at d3 also expressed CD43 (10.2±3.8%, *n*=5), implying a modest haemogenic capacity for these cells (Fig. 1G). In summary, these data demonstrated expression of SOX17 in extra-embryonic, yolk sac-like endothelium, which appeared at the same time as CD43^+^ haematopoietic cells, and before RUNX1C expression in a subset of the nascent blood cells.

### SOX17-negative endothelium is the major source of the first human blood cells

In order to determine the relationship between the different cell types during differentiation, we flow sorted d2 blast colonies after 3 days in methylcellulose into endothelial (SOX17^+^CD34^+^CD43^−^ and SOX17^−^CD34^+^CD43^−^, hereafter denoted d3 SOX17^+^ENDO and d3 SOX17^−^ENDO) and haematopoietic (CD34^+^CD43^+^ and CD34^−^CD43^+^) populations (Fig. 1H and Fig. S1A). Re-culturing the haematopoietic populations yielded predominantly GYPA^+^ erythroid cells (76.1±4.1%, *n*=3) from the more mature CD34^−^CD43^+^ fraction, while the CD34^+^CD43^+^ fraction yielded more RUNX1C^+^ non-erythroid (myeloid and megakaryocytic) cells (65.7±6.8%, *n*=3) (Fig. 1I and Fig. S1B), suggesting an erythroid bias in the earliest blood cells produced from blast colonies. The d3 SOX17^+^ENDO cells generated few CD43^+^ cells (12.8±8.6%, *n*=3) (Fig. 1I), whereas the d3 SOX17^−^ENDO population generated a very high proportion of CD43^+^ blood cells (87.8±0.9%, *n*=3) and infrequent SOX17^+^ endothelial cells (7.3±1.1%, *n*=3) (Fig. 1I).

Both d3 SOX17^+^ENDO and d3 SOX17^−^ENDO cells formed endothelial-like networks in Matrigel within 24 h (Fig. 1J,K). The SOX17^+^ cultures retained expression of the mCHERRY reporter, while most of the SOX17^−^ cells remained mCHERRY negative, suggesting that the allocation of cells to a SOX17^+^ fate was largely complete by d3 in methylcellulose (Fig. 1J,K). Continuing the cultures for a further 24 h in the presence of stem cell factor generated many haematopoietic cells from SOX17^−^ENDO cells but only infrequent foci of blood cells in SOX17^+^ENDO cultures (Fig. 1L,M), consistent with the results of re-culture experiments (Fig. 1I).

The frequency of haematopoietic progeny from SOX17^−^ENDO cells, sorted after 2 days of methylcellulose culture, was determined by limit dilution analysis and compared with the haemogenic frequency of SOX17^+^ENDO and SOX17^−^ENDO cells sorted from d3 of methylcellulose differentiation (Fig. 1H,N and Table S1). The high frequency of haemogenic SOX17^−^ENDO cells isolated at d2 or d3 of methylcellulose culture (both 1:11) contrasted with the low frequency (1:2480) observed in d3 SOX17^+^ENDO cells (Fig. 1N and Table S1).

**Fig. 2.**
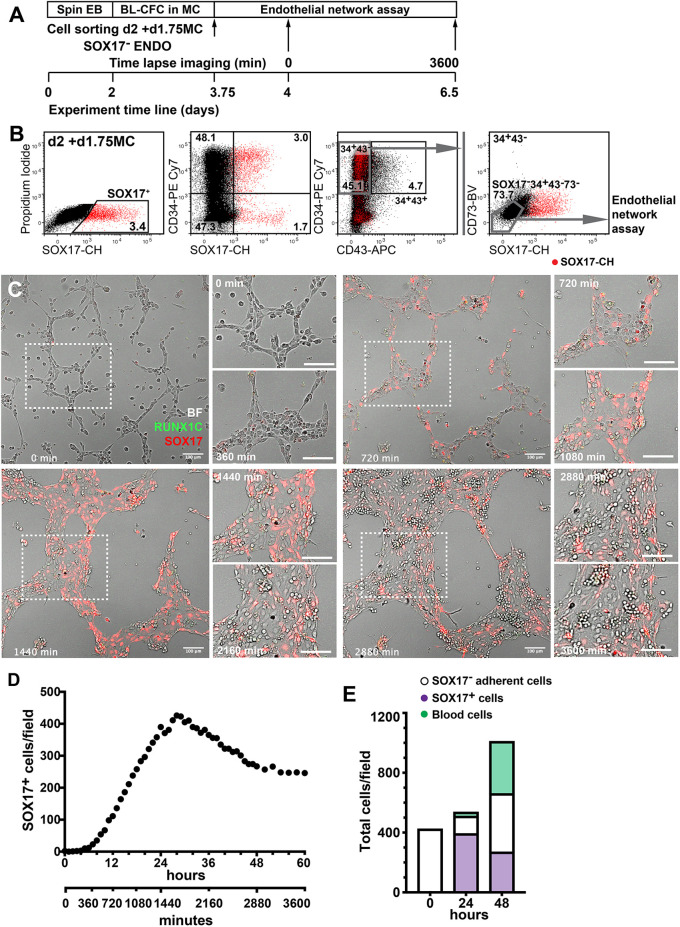
**SOX17-negative endothelium gives rise to SOX17-positive endothelium.** (A) Experimental outline. After 2 days of differentiation as spin EBs, cells were harvested and plated in MC in a BL-CFC assay. After 1.75 days in MC, SOX17^−^ ENDO cells were sorted and plated in a Matrigel endothelial tube assay. Six hours after seeding cells, time-lapse imaging of cells commenced in a humidified chamber at 37°C with 5% CO_2_ in air. EB, embryoid body; BL-CFC, blast colony-forming cell; MC, methylcellulose. (B) Gating strategy outlining the sorting of SOX17^−^ENDO cells from MC at d2+d1.75. The two left-hand panels show the expression of SOX17 and of CD34 at this time point. The right-hand panels show the sequential gating strategy used to sort the SOX17^−^ENDO (SOX17^−^CD34^+^CD43^−^CD73^−^) endothelial cells that were seeded into the endothelial tube assay. (C) Images taken from the time lapse series at illustrative time points from 0 to 3600 min as indicated on each image. Boxed areas and matching fields from intermediate time points are shown at higher magnification. The first faintly red fluorescent SOX17^+^ cells are seen from 360 min (6 h) and are obvious from 720 min (12 h) onwards. Blood cells appear from 2160 min (36 h). Scale bars: 100 µm. (D) Graph plotting the number of fluorescent SOX17 cells in the imaged field at hourly intervals, showing a rapid emergence of SOX17^+^ cells from 360 min to 1440 min, before a reduction and plateauing of numbers. The field area was 0.9 mm^2^. (E) Bar graph detailing the number of SOX17^−^ and SOX17^+^ cells and the number of morphologically classified haematopoietic cells at the onset, and at 24 h and 48 h after the onset of time lapse imaging. There is little increase in cell number over the initial 24 h, although there is a rapid rise in the number of SOX17^+^ cells. See also Movie 1 and Fig. S2.

In order to explore the developmental relationship between SOX17^−^ and SOX17^+^ endothelium, we performed live cell imaging over 65 h of d2 SOX17^−^ENDO cells sorted from methylcellulose cultures and re-plated into a Matrigel endothelial network assay (Movie 1, Fig. 2 and Fig. S2). It can be seen from time-lapse images taken at 10 min intervals (Movie 1 and Fig. 2C) that the endothelia were initially SOX17^−^, and that individual SOX17^−^ cells began to acquire expression of the SOX17 reporter after 6 h of observation. Importantly, mCHERRY expression was acquired by 24-28 h, and during this period there was little increase in cell numbers, precluding division of any rare contaminating SOX17^+^ cells present at the onset of the culture as the reason for the increase in SOX17^+^ cell numbers (Fig. 2D). After this period, the number of blood cells rapidly increased and the number of SOX17^+^ cells decreased a little and stabilised. Similar kinetics of mCHERRY reporter expression were observed in a second experiment that was not subjected to time-lapse imaging (Fig. S2). These data strongly support the premise that d3 SOX17^+^ENDO derives from the same d2 SOX17^−^ENDO precursor population that also exhibits high haemogenic activity (Fig. 1N).

As we had observed that a small percentage of SOX17^+^ENDO cells developed into CD43^+^ haematopoietic cells (Fig. 1G), we compared them with SOX17^−^ENDO derived CD43^+^ cells. We sorted haematopoietic and endothelial cells at d3 of methylcellulose culture that were either SOX17^+^ or SOX17^−^, confirming that the SOX17^+^ cells expressed higher levels of *mCHERRY* and of *SOX17*, and providing reassurance that the appearance of SOX17^+^CD43^+^ cells was not the consequence of imperfect sorting (Fig. S3A,B). The expression of haematopoietic genes [*RUNX1*, *GFI1*, *SPI1* (previously known as *PU.1*), *KLF1* and *GATA1*] was higher in the d3 SOX17^−^ENDO endothelial cells, probably reflecting their greater haemogenic capacity (Fig. S3B). The CD34^+^CD43^+^ derivatives of both SOX17^+^ and SOX17^−^ endothelium expressed similar levels of haematopoietic transcription factors and globin genes, and displayed similar morphology, frequency and distribution of colony-forming cells (Fig. S3B-G). These data suggest that, in human extra-embryonic, yolk sac-like cultures, blood cells predominantly derive from *SOX17*^−^ endothelium, with a very small proportion of phenotypically similar cells arising from precursors that are *SOX17*^+^.

### Identification of distinct endothelial and haematopoietic subsets of differentiating blast colonies

**Fig. 3.**
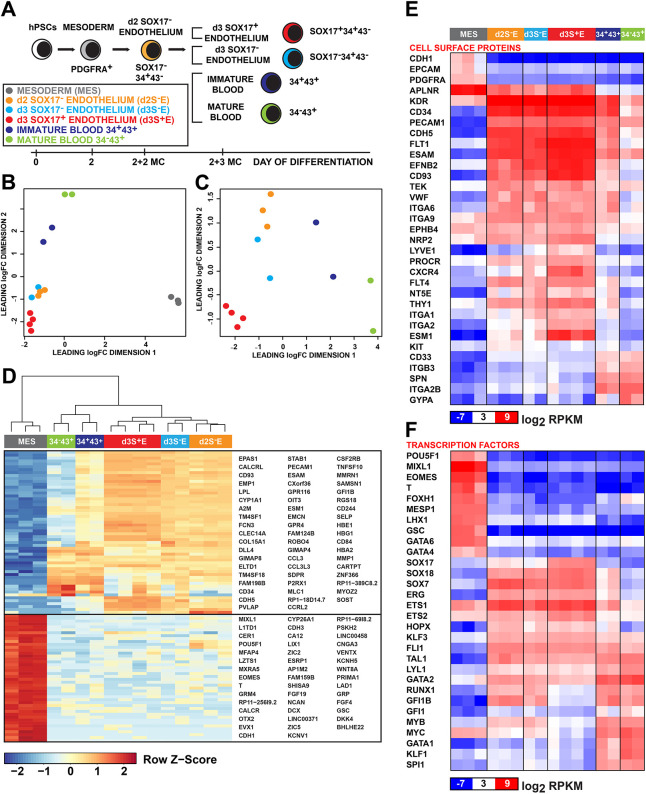
**Transcriptional profiling reveals discrete endothelial and haematopoietic populations.** (A) Flow-sorted fractions (shown in black text) collected for RNA-seq analysis. D2 cells sorted from embryoid bodies (EBs): PDGFRα^+^ mesoderm (MES, grey). D2 MES cells cultured for 2 days in methylcellulose (2+2 MC): SOX17^−^ENDO[CD34^+^CD43^−^] (d2S^−^E, orange). D2 MES cells cultured for 3 days in methylcellulose (2+3MC): SOX17^+^ENDO[CD34^+^CD43^−^] (d3S^+^E, red), SOX17^−^ENDO[CD34^+^CD43^−^] (d3S^−^E, cyan), 34^+^43^+^ immature blood (blue) and 34^−^43^+^ mature blood (green) populations. (B,C) Multidimensional scaling plots of sorted populations showing (B) mesoderm separation from endothelial and haematopoietic populations, and (C) excluding mesoderm to demonstrate clustering of haematopoietic (34^−^43^+^, 34^+^43^−^), SOX17^−^ENDO (d2S^−^E, d3S^−^E) and SOX17^+^ENDO (d3S^+^E) fractions. (D) Unsupervised hierarchical clustering of RNA-seq data showing that the top 100 differentially expressed genes define mesoderm and haematopoietic/endothelial populations. Genes included in the two clusters are shown. Scale indicates row Z-score. See also Table S3. (E,F) Heatmaps comparing selected genes differentially expressed between sorted populations categorised as (E) cell surface proteins and (F) transcription factors. Scale indicates log_2_ reads per kilobase per million (RPKM). See also Fig. S4 and Table S4 for details of differentially expressed genes.

We compared the transcriptional profiles of d2 PDGFRα^+^ mesoderm, d2 and d3 methylcellulose SOX17^−^ENDO cells, d3 methylcellulose SOX17^+^ENDO cells, and d3 methylcellulose CD34^+^CD43^+^ and CD34^−^CD43^+^ haematopoietic populations (Fig. 3A-C and Table S2). Mesodermal cells and endothelial/haematopoietic samples clustered into separate cell populations (Fig. 3D). Differential gene expression analysis revealed that upregulated genes in mesoderm were enriched for gene ontology (GO) terms associated with embryo development (GO:0009790) and gastrulation (GO:0007369), while the endothelial/haematopoietic populations were enriched for leukocyte (GO:0050900) or vascular-related (GO:0001944) genes (Table S3). Although very few genes were differentially expressed between d2 and d3 SOX17^−^ENDO cells, several thousand genes were differentially expressed between CD34^+^CD43^+^ and CD34^−^CD43^+^ haematopoietic cells and d2 SOX17^−^ENDO cells (Fig. S4A,B and Table S4).

Patterns of differentially expressed genes between haematopoietic and endothelial cells were also consistent with cells segregating into distinct *SOX17*-expressing endothelium or CD34^+^CD43^+^ haematopoietic fates during blast colony differentiation (Fig. S4C). Specifically, 914/1062 (86.1%) genes upregulated in d3 SOX17^+^ENDO were downregulated in CD34^+^CD43^+^ blood cells, and 711/954 (74.5%) of genes downregulated in d3 SOX17^+^ENDO were upregulated in CD34^+^CD43^+^ blood cells (Fig. S4D and Table S4). Similarly, 851/1028 (82.8%) genes upregulated in CD34^+^CD43^+^ were downregulated in d3 SOX17^+^ENDO cells and 1647/1898 (86.8%) of genes downregulated in CD34^+^CD43^+^ were upregulated in d3 SOX17^+^ENDO cells (Fig. S4E and Table S4). The data can be summarised to state that the same genes upregulated during the transition from d2 SOX17^−^ENDO to d3 SOX17^+^ENDO are downregulated in the transition to CD34^+^CD43^+^, and vice versa. These analyses argue for the presence of a binary ‘switch’ that is active in the d2 SOX17^−^ENDO cells and will lead to either a haematopoietic or endothelial fate; they are also consistent with a haemangioblast-like function of these cells.

Examination of the specific genes expressed in the sorted populations revealed there was selective expression of cell surface proteins (including *CDH1*, *EPCAM* and *PDGFRA*) and transcription factors (including *POU5F1*, *MIXL1*, *EOMES* and *T*) associated with the primitive streak stage of development (Davis et al., 2008; Hirst et al., 2006) in the mesoderm cells (Fig. 3E,F). Consistent with our previous findings (Ng et al., 2016), *HOXB*, but not *HOXA*, genes were upregulated in these extra-embryonic, yolk sac-like endothelial populations (Fig. S4F).

Two surface markers identifying blast colony-forming cells, *KDR* (Kennedy et al., 2007) and *APLNR* (Yu et al., 2012), were expressed in the mesoderm and in their endothelial progeny (Fig. 3E). There was a high concordance in the expression of endothelial cell surface genes (including *CD34*, *PECAM1*, *CDH5*, *FLT1*, *ESAM*, *EFNB2* and *CD93*) and transcription factors (including *SOX7*, *SOX18*, *ERG*, *ETS1*, *ETS2*, *HOPX* and *FLI1*) in d2 and d3 SOX17^−^ and d3 SOX17^+^ENDO samples (Fig. 3E,F). In addition to upregulated *SOX17* expression, we observed reduced expression of cell cycle genes and the proliferation-related transcription factors *MYB* and *MYC* in the d3 SOX17^+^ENDO cells, suggesting that these cells were more quiescent, possibly mediated by higher levels of NOTCH signalling (Mack and Iruela-Arispe, 2018) (Fig. 3F and Fig. S4F). Expression of a number of genes distinguished the CD43^+^ haematopoietic fractions from their endothelial counterparts, including the surface-expressed *SPN* (previously known as CD43), *ITGA2B* (previously known as CD41), *ITGB3* (previously known as CD61), *CD33* and the transcription factors *GATA1*, *KLF1* and *SPI1* (*PU.1*) (Fig. 3E,F). The acquisition of CD43 expression was also associated with a simultaneous downregulation of endothelial cell surface markers (including *APLNR*, *CDH5*, *FLT1*, *ESAM*, *EFNB2* and *CD93*) and transcription factors (including *SOX7*, *SOX17*, *SOX18*, *ERG*, *ETS1*, *ETS2* and *HOPX*) (Fig. 3E,F).

Notably, there was variation in expression between *RUNX1* and *GFI1B* in the endothelial populations (Fig. 3F). Higher levels of *RUNX1* and *GFI1B* expression in the d2 and d3 SOX17^−^ENDO cells correlated with a high capacity to form haematopoietic cells, while low levels of *RUNX1* and *GFI1B* in d3 SOX17^+^ENDO marked a largely non-haemogenic endothelium. In order to explore the role of these factors in dictating haemogenic capacity, we characterised differentiation in cell lines in which they were deleted or inhibited.

### *RUNX1* is required for blast colony development

**Fig. 4.**
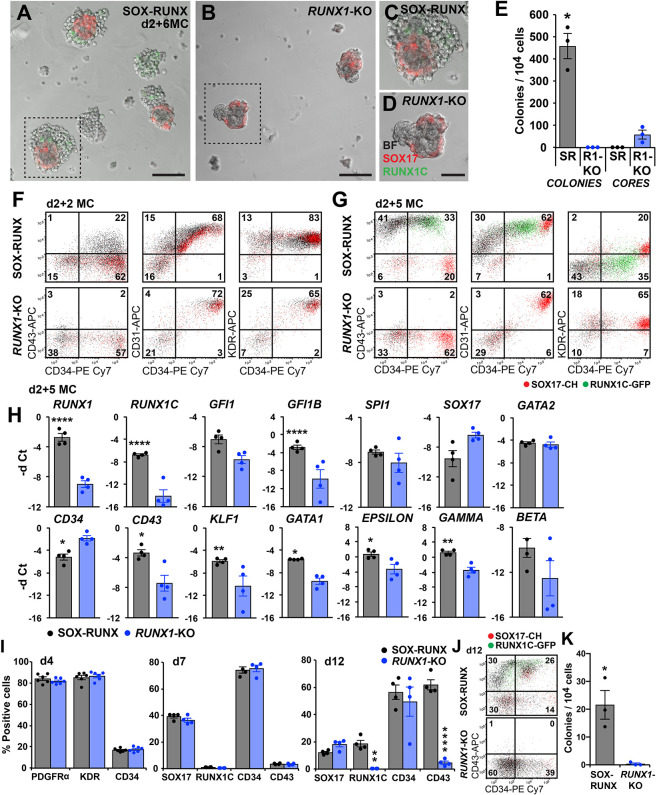
**Blast colony development requires *RUNX1*.** (A-D) Blast colony cultures after 6 days in methylcellulose (d2+6MC), showing (A,C) blast colonies generated from SOX-RUNX cultures and (B,D) SOX17-expressing vascular cores from *RUNX1*-KO cultures. Scale bars: 100 µm (*n*=9 experiments). (E) Blast colonies were observed only from SOX-RUNX (SR) cultures and endothelial cores only from *RUNX1*-KO (R1-KO) cultures (mean±s.e.m., *n*=3 experiments). *P<0.02 compared with *RUNX1*-KO (unpaired Student's *t*-test). (F,G) Flow cytometry plots of d2 (d2+2MC) and d5 (d2+5MC) SOX-RUNX and *RUNX1*-KO methylcellulose cultures illustrating the absence of CD43^+^ blood cells in *RUNX1*-KO cultures (*n*=4 experiments at d2+2 MC; *n*=3 experiments at d2+5 MC). Data values are provided in the text. Day 8 (d2+8MC) plots shown in Fig. S5D. (H) Relative gene expression [negative delta (−d) Ct] for selected haematopoietic, genes in d2+5 MC *RUNX1*-KO cultures compared with SOX-RUNX control cultures (mean±s.e.m., *n*=4 experiments). *P<0.03, **P<0.002, ****P<0.0001 compared with SOX-RUNX (two-way ANOVA, Holm Sidak's multiple comparisons test). (I) Analysis of mesoderm (PDGFRα and KDR), haematopoietic (CD34, CD43) and reporter (SOX17 and RUNX1C) genes at d4, d7 and d12 of differentiation of SOX-RUNX and *RUNX1*-KO cultures (mean±s.e.m. for *n*=6 experiments at d4; *n*=4 experiments at d7, d12). **P<0.002, ****P<0.0001 compared with SOX-RUNX (two-way ANOVA, Holm Sidak's multiple comparisons test). (J) Flow cytometry plots illustrating the absence of CD43^+^ and RUNX1C^+^ blood cells in *RUNX1*-KO at d12 compared with the SOX-RUNX control (*n*=4 experiments). (K) No d7 haematopoietic colonies were observed in *RUNX1*-KO methylcellulose cultures (mean±s.e.m., *n*=3 experiments). *P=0.048 compared with SOX-RUNX control (unpaired Student's *t*-test).

To examine whether *RUNX1* is a key driver of the EHT in human extra-embryonic, yolk sac-like haematopoiesis, we generated *RUNX1*-null hPSCs (denoted *RUNX1*-KO) by excising part of the DNA-binding domain of *RUNX1* in SOX-RUNX cells (see Materials and Methods and Fig. S5A-C,G). In blast colony assays, *RUNX1*-KO cultures formed vascular cores that expressed the *SOX17* reporter, but did not obviously generate haematopoietic cells (Fig. 4A-E). Flow cytometry analyses confirmed a failure of CD43^+^ blood cells to increase in *RUNX1*-KO cultures (19.9±3.1%, SOX-RUNX; 2.9±0.3%, *RUNX1*-KO; *n*=4, *P*<0.01, Student's *t*-test) at d2 of methylcellulose culture (Fig. 4F). By d5 (Fig. 4G and Fig. S5E) and d8 (Fig. S5D,E) of methylcellulose culture, more striking reductions in CD43^+^, GYPA^+^ and RUNX1C^+^ cells were noted in *RUNX1*-KO compared with SOX-RUNX cultures. Gene expression studies confirmed the downregulation of *RUNX1*, *RUNX1C* and *GFI1B*, with similarly reduced expression of erythroid lineage genes (*GATA1*, *KLF1*, *ε-GLOBIN* and *γ-GLOBIN*) and increased expression of endothelial *SOX17* and *CD34* (Fig. 4H and Fig. S5F).

### Primitive erythroid cell generation in *RUNX1*-KO cells is *GFI1* dependent

**Fig. 5.**
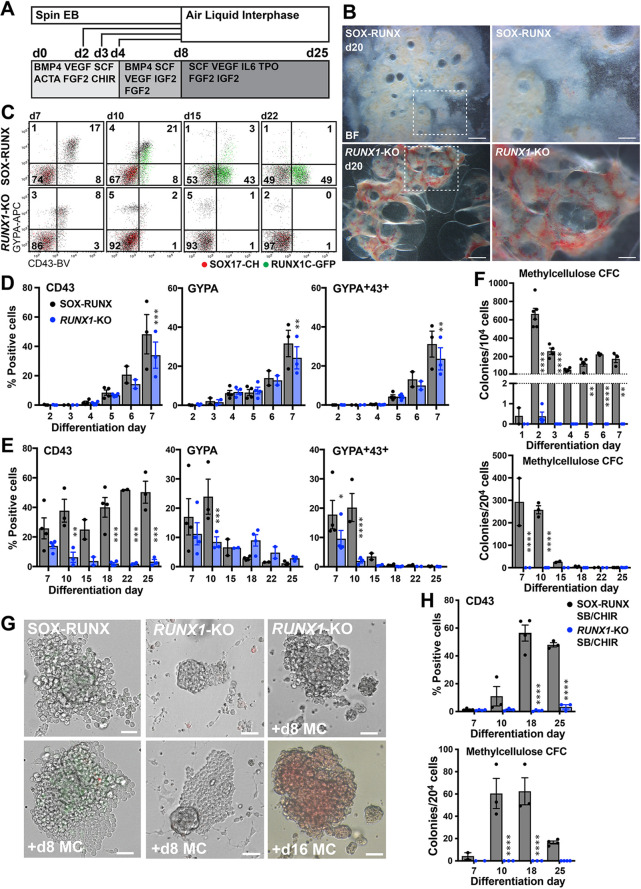
***RUNX1*-knockout cultures exclusively generate primitive erythroid lineages.** (A) Air-liquid interphase differentiation protocol. (B) *RUNX1*-KO embryoid bodies (EBs) grown on air-liquid interphase (ALI) were more haemoglobinised than SOX-RUNX controls by d20 (d8+12 ALI). Scale bars: 1000 µm (left); 400 µm (right) (*n*=4 experiments). (C) *RUNX1*-KO cultures maintained GYPA^+^ erythroid cells but failed to develop RUNX1C^+^CD43^+^ myeloid cells (*n*=2-4 experiments per time point). (D) Similar early blood cell differentiation in *RUNX1*-KO and SOX-RUNX cultures, with differences observed at day 7 (mean±s.e.m., *n*=2-4 experiments per time point). **P<0.01, ***P<0.001 (two-way ANOVA, Holm Sidak's multiple comparisons test). (E) SOX-RUNX cultures developed more CD43^+^ myeloid cells than *RUNX1*-KO cells (mean±s.e.m., *n*=2-4 experiments per time point). *P<0.02, **P<0.01, ***P<0.001, ****P<0.0001 (two-way ANOVA, Holm Sidak's multiple comparisons test). (F) Clonogenic frequency peaked at d2 in SOX-RUNX methylcellulose cultures, while *RUNX1*-KO cultures generated only rare erythroid colonies at d2. Upper and lower panels represent results from two separate series of experiments (mean±s.e.m., *n*=2-6 experiments per time point). **P<0.01, ****P<0.0001 (two-way ANOVA, Holm Sidak's multiple comparisons test). (G) Haematopoietic colonies arising from d2 EBs in SOX-RUNX cultures included RUNX1C^+^ myeloid cells, while the rare *RUNX1*-KO colonies comprised only erythroid cells emerging from a vascular core. The far right panel displays the same *RUNX1*-KO colony imaged at d8 in methylcellulose (+d8 MC) and after +d16 MC to demonstrate haemoglobinisation (*n*=6 experiments). (H) Intra-embryonic SB CHIR (S/C) differentiated SOX-RUNX cultures consistently generated CD43^+^ blood cells and colonies in methylcellulose, in comparison with *RUNX1*-KO. Upper and lower panels represent results from the same experimental series (mean±s.e.m., *n*=2-4 experiments per time point). ****P<0.0001 (two-way ANOVA, Holm Sidak's multiple comparisons test).

The presence of abundant nucleated erythroid cells in *Runx1* knockout mouse embryos at E12.5 (Okuda et al., 1996; Wang et al., 1996) argues that the initial wave of yolk sac erythroid differentiation remains intact, although we were initially unable to detect expansion of CD43^+^ blood cells in human *RUNX1*-KO cells after d7 (Fig. 4I,J) or colony-forming cells in the d7 *RUNX1*-KO differentiation cultures (Fig. 4K). We then added FGF2 and a low concentration of CHIR from the onset of differentiation (Fig. 5A), because previous studies have shown that WNT agonists synergise with BMP4 to promote differentiation of haematopoietic mesoderm (Gertow et al., 2013), and that early haematopoietic colonies are FGF2 dependent (Choi et al., 2012; Yu et al., 2012). These modifications, combined with culturing EBs at an air-liquid interface, led to the appearance of haemoglobinised cells, more prominent in the *RUNX1*-KO cultures, after d15 of differentiation (Fig. 5A,B). There were more CD43^+^ cells in both SOX-RUNX and *RUNX1*-KO cultures from d5-d7 under these conditions, with most cells co-expressing GYPA (compare Fig. 5C,D with Fig. 4I).

After d7, an increasing proportion of SOX-RUNX cells expressed CD43, often associated with RUNX1C, and downregulated GYPA. In contrast, no new CD43^+^ cells appeared in the *RUNX1*-KO cultures. These cells downregulated CD43, but retained high levels of GYPA expression, consistent with adoption of an erythroid fate (Fig. 5C,E). These data indicate that *RUNX1* is not required for the generation of the first CD43-expressing cells that subsequently differentiate only to erythroid cells. This is consistent with observations in *Runx1*-null embryos and differentiated *Runx1*-null mouse ES cells, in which all myeloid cells are absent (Lacaud et al., 2002; Okuda et al., 1996; Wang et al., 1996). We compared the frequency of colony-forming cells in differentiating SOX-RUNX and *RUNX1*-KO cultures. In SOX-RUNX cells, BL-CFC peaked at d2 of differentiation, as noted previously (Fig. 1D), followed by a wave of primarily erythroid colonies at d6-d7 (Fig. 5F). However, in *RUNX1*-KO cultures, the only clonogenic cells detected were at d2, when a small number of erythroid colonies were observed arising from vascular cores when cells were cultured at high density in methylcellulose (Fig. 5F,G).

Supporting evidence for the erythroid restriction of *RUNX1*-KO haematopoiesis was provided by RNA-seq analysis at d6 of differentiation. At this early time point there were only 17 differentially expressed genes between the parental SOX-RUNX and the *RUNX1*-KO cultures (Tables S5 and S7). Notably, 5 out of the 10 genes downregulated in the d6 *RUNX1*-KO cell line are expressed in megakaryocytes, myeloid cells or B cells (*HDC*, *GCSAML*, *PLEK*, *RGS18* and *TSPAN33*) consistent with the hypothesis that differentiation to non-erythroid lineages is compromised. Conversely, three out of seven genes with increased expression in *RUNX1*-KO cells are expressed in erythroid cells (*SLC4A1*, *HBA2* and *HEMGN*), arguing for a complementary increase in erythroid differentiation, as indeed was observed under these modified differentiation conditions.

**Fig. 6.**
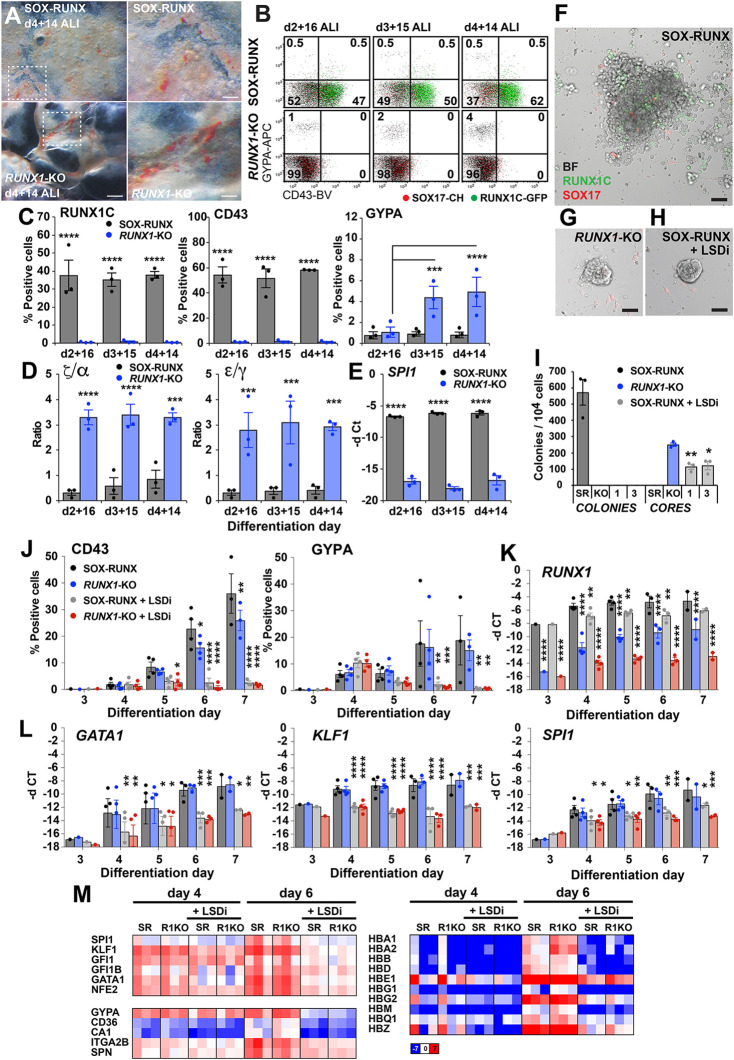
**LSD1 inhibition blocks extra embryonic erythroid differentiation from *RUNX1*-KO cultures.** (A) Day 4 embryoid bodies (EBs) cultured for 14 days on air-liquid interphase (ALI) (d4+14 ALI). *RUNX1*-KO cultures were more extensively haemoglobinised compared with SOX-RUNX controls (*n*=3 experiments). Scale bars: 1000 µm (left); 400 µm (right). (B) *RUNX1*-KO EBs cultured on air-liquid interphase for 14 to 16 days retained a higher number of GYPA^+^ erythroid cells compared with the SOX-RUNX controls (*n*=3 experiments). (C) Analysis of the experiments in B illustrating the predominance of RUNX1C^+^ and CD43^+^ cells in SOX-RUNX cultures (mean±s.e.m., *n*=3 experiments). ***P<0.001 and ****P<0.0001 (two-way ANOVA, Holm Sidak's multiple comparisons test). (D,E) Gene expression analysis of experiments shown in B,C, highlighting (D) higher ratios of ζ/α and ε/γ globin in *RUNX1*-KO cultures, while (E) SOX-RUNX cultures expressed higher levels of the myeloid gene *SPI1* (mean±s.e.m., *n*=3 experiments). ***P<0.001 and ****P<0.0001 (two-way ANOVA, Holm Sidak's multiple comparisons test). (F-H) Images of (F) d2+9 haematopoietic SOX-RUNX colonies and similar vascular core colonies in (G) *RUNX1*-KO and (H) SOX-RUNX LSD1 inhibitor (LSDi)-treated cultures. Scale bars: 50 µm (*n*=3 experiments). (I) Blast colonies (colonies) were confined to SOX-RUNX cultures and vascular cores (cores) in *RUNX1*-KO and LSDi 1 µM (1) and 3 µM (3) supplemented SOX-RUNX cultures (mean±s.e.m., *n*=3 experiments). *P<0.022, **P<0.0037 compared with *RUNX1*-KO cores (unpaired Student's *t*-test). (J) d3-d7 LSDi-treated SOX-RUNX EBs fail to generate CD43^+^ or GYPA^+^ cells (mean±s.e.m., *n*=1 experiment for d3; *n*=3 or 4 for d4-7). *P<0.05, **P<0.01, ***P<0.001, ****P<0.0001 compared with SOX-RUNX cells (two-way ANOVA, Dunnett's multiple comparisons test). (K,L) Gene expression analysis of the experiments in J showing (K) *RUNX1* reduction in LSDi-treated cultures and loss in *RUNX1-*KO cultures and (L) LSDi downregulation of *GATA1*, *KLF1* and *SPI1* (mean±s.e.m., *n*=1 experiment for d3, *n*=2 experiments d7, *n*=3-4 experiments for d4–d6). *P<0.05, **P<0.01, ***P<0.001, ****P<0.0001 (two-way ANOVA, Dunnett's multiple comparisons test). (M) Heatmaps of selected RNA seq data from d4 and d6 SOX-RUNX (SR), RUNX1-KO (R1KO) cultures showing downregulation of gene expression with LSDi treatment. Scale indicates log_2_ reads per kilobase per million (RPKM). See also Fig. S6D.

We confirmed the crucial role of *RUNX1* in specifying intra-embryonic, AGM-like haematopoiesis (Chen et al., 2009b; North et al., 2002), demonstrating that CD43^+^ blood cells were not generated in *RUNX1*-KO cultures, and colony formation was not observed, when cultures were differentiated under conditions that facilitated the emergence of AGM-like blood lineages (Ng et al., 2016) (Fig. 5H). In order to determine the optimum differentiation day at which to transfer embryoid bodies to air-liquid interface cultures for erythroid development, transfers at d2, d3 and d4 were compared (Fig. 6A). Haemoglobinised clusters were most evident in *RUNX1*-KO cells cultured on air-liquid interface from d4, with lesser amounts in SOX-RUNX cultures (Fig. 6A). Flow cytometry confirmed the higher percentage of GYPA-expressing cells in the *RUNX1*-KO cultures, and demonstrated that RUNX1C and CD43 expression was confined to the SOX-RUNX cultures (Fig. 6B,C). Analysis of globin expression revealed a higher ratio of ζ/α and ε/γ chains in differentiated *RUNX1*-KO cells, indicating the developmentally earlier phenotype of the erythroid cells in these cultures (Fig. 6D). Mirroring the pattern of *RUNX1C* expression, PCR analysis of *SPI1* (*PU.1*) revealed higher expression in the SOX-RUNX cultures (Fig. 6E).

*RUNX1* and *SPI1* are expressed in erythroid progenitor cells, but their levels decline with erythroid maturation. The decline in *RUNX1* is the major driver of the reduction in *SPI1* expression (Willcockson et al., 2019). Both *RUNX1* and *SPI1* repress key erythroid transcription factors, exemplified by *KLF1* (Kuvardina et al., 2015), and the enforced expression of either gene prevents terminal erythroid differentiation (Willcockson et al., 2019). We hypothesise that in the absence of *RUNX1* driving myeloid and megakaryocytic differentiation, *SPI1* levels also eventually fall, and the wave of GYPA^+^CD43^+^ cells seen at d7 (Fig. 5C) defaults entirely to erythroid lineage differentiation. This may explain the greater quantity of primitive erythroid cells generated from *RUNX1*-KO cells.

The *Runx1*-target genes *Gfi1* and *Gfi1b* (*Gfi1*/*1b*) are expressed in haemogenic endothelia in both the E11.5 dorsal aorta and in the E9.5 yolk sac in the mouse (Thambyrajah et al., 2016b). Gfi1/1b form multi-protein complexes that include the co-repressor Rcor1 (CoREST), the histone demethylase Kmd1a (Lsd1), and the histone deacetylases Hdac1 and Hdac2 (Saleque et al., 2007). It has been argued that *Runx1* expression in haemogenic endothelium induces *Gfi1*/*1b*, which bind to regulatory sequences of endothelial genes, and recruit the CoREST complex that then silences the endothelial program (Lancrin et al., 2012; Thambyrajah et al., 2016b). Inhibition of Lsd1 has been shown to phenocopy the endothelial to haematopoietic transition block observed with deletion of *Gfi1*/*1b* (Thambyrajah et al., 2016a). When inhibitors of LSD1 (denoted LSDi) were included in blast colony assays, all blood cell formation was lost, and only cores of endothelial and stromal cells remained, similar to findings with *RUNX1*-KO cultures (compare Fig. 6F-I with Fig. 4A-E). The endothelium generated under LSDi conditions in the blast colony assay was very similar to endothelium produced by the *RUNX1*-KO cells and the SOX-RUNX parental cell line. After 10 days of differentiation in methylcellulose, there were very few endothelial cells (CD34^+^CD31^+^KDR^+^CD43^−^) in the SOX-RUNX cultures (<1%) and these were >95% SOX17^+^ (Fig. S6A,B). In the *RUNX1*-KO and the LSDi-treated SOX-RUNX cultures, ∼30-40% of the viable cells were SOX17^+^ endothelial cells (Fig. S6A,B).

However, LSDi-treated cultures, in contrast to *RUNX1*-KO lines, failed to generate or maintain CD43^+^ or GYPA^+^ cells (Fig. 6J). PCR analysis indicated that levels of *RUNX1* transcripts were lower in cultures treated with LSDi (compare SOX-RUNX with and without LSDi in Fig. 6K and Fig. S5G), suggesting that *GFI1*/*1B* may be a regulator, as well as a target, of *RUNX1*. Other haematopoietic transcription factors [*GATA1*, *KLF1* and *SPI1* (*PU.1*)] were also significantly downregulated by LSD1 inhibition (Fig. 6L), consistent with the endothelial-to-haematopoietic transition block. These differences in transcription factor expression in LSDi-treated cultures were seen from d4 of differentiation, pre-dating the emergence of CD43^+^ cells, and therefore excluding major differences in the cellular composition of the cultures as an explanation for this observation. Expression of these genes was similar between SOX-RUNX and *RUNX1-*KO cells, consistent with a primary role of Runx1 in reorganisation, rather than transcriptional regulation, of lineage-specific transcription factor assemblies (Lichtinger et al., 2012; Thambyrajah et al., 2016b) (Fig. 6L). RNA-seq analysis in d4 and d6 LSDi-treated cultures indicated a downregulation in expression of erythroid lineage genes, associated with gene ontology terms oxygen transport (GO:0015671) and haemoglobin complex (GO:0005833) (Fig. 6M, Figs S5H and S6C,D, and Tables S5 and S6). Genes with increased expression in LSDi most notably included those responsive to the HDAC inhibitor panobinostat (Fig. 6M, Fig. S6C,D and Tables S5 and S6). This is consistent with the inhibition of the HDAC containing CoREST complex by LSDi, and with the similar effects on murine haematopoiesis observed between Lsd1 and Hdac inhibition (Thambyrajah et al., 2018).

### The human blast colony assay detects predominantly *RUNX1*-dependent haematopoiesis

*RUNX1* is required for the formation of virtually all haematopoietic cells detected in the blast colony assay (Fig. 4), suggesting that this primarily reads out progenitor cells similar to mouse yolk sac EMPs, which are also *Runx1* dependent (Tober et al., 2013). One prediction of this hypothesis is that blast colonies should generate granulocytes, a lineage not observed during the first wave of extra-embryonic yolk-sac blood formation (Palis et al., 1999). To test this, we differentiated blast colony-forming cells in the presence of growth factors that preferentially support erythroid, macrophage or granulocytic cells (Fig. S7A). May-Grünwald-Giemsa stained cytospin preparations documented that erythroid cells were restricted to cultures supplemented with EPO, macrophages were dependent upon M-CSF, and maturing neutrophils and eosinophils dominated cultures supplemented with G-CSF and GM-CSF (Fig. S7B). These lineage assignments were supported by flow cytometry, showing expression of GYPA on erythroid cells, CD14 expression on macrophages and RUNX1C in granulocytes (Fig. S7C,D). Finally, PCR analysis confirmed globin, *KLF1* and *GATA1* expression in erythroid cells, *CSF1R* and *SPI1* in macrophages, and *EPX*, *SPI1* and *GATA1* in granulocytes (Fig. S7E). Taken together, these data support our hypothesis that the human blast colony assay reads out *RUNX1*-dependent extra-embryonic, yolk sac-like cells with a broad myeloid potential similar to mouse yolk sac EMPs.

## DISCUSSION

We have modelled extra-embryonic human haematopoiesis and dissected the role of the transcription factor *RUNX1* in analyses facilitated by the use of a reporter line in which GFP reported cells expressing the haematopoietic specific, *C* isoform, of *RUNX1* and mCHERRY, expressed from the *SOX17* locus, marked endothelium. We showed that d2 SOX17^−^ ENDO cells that expressed *RUNX1* and *GFI1/1B* functioned as a haemangioblast-like population. Continued expression of *RUNX1* and *GFI1/1B* was correlated with the emergence of extra-embryonic, yolk sac-like blood cells, while the upregulation of *SOX17* expression and extinction of *RUNX1* and *GFI1/1B* led to the development of SOX17^+^ endothelial cells. This complementary expression of *RUNX1* and *SOX17* in blood and endothelial cells, captured in the RNA-seq analysis shown in Fig. 3, is reminiscent of the expression pattern of these transcription factors in the human intra-embryonic AGM, where endothelial cells transitioning to form intra-aortic haematopoietic clusters expressed lower levels of SOX17 and increasing RUNX1 (Bos et al., 2015). Our data suggest that a similar reciprocal regulation of *RUNX1* and *SOX17* expression occurs in our model of human extra-embryonic yolk sac-like haematopoiesis.

**Fig. 7.**
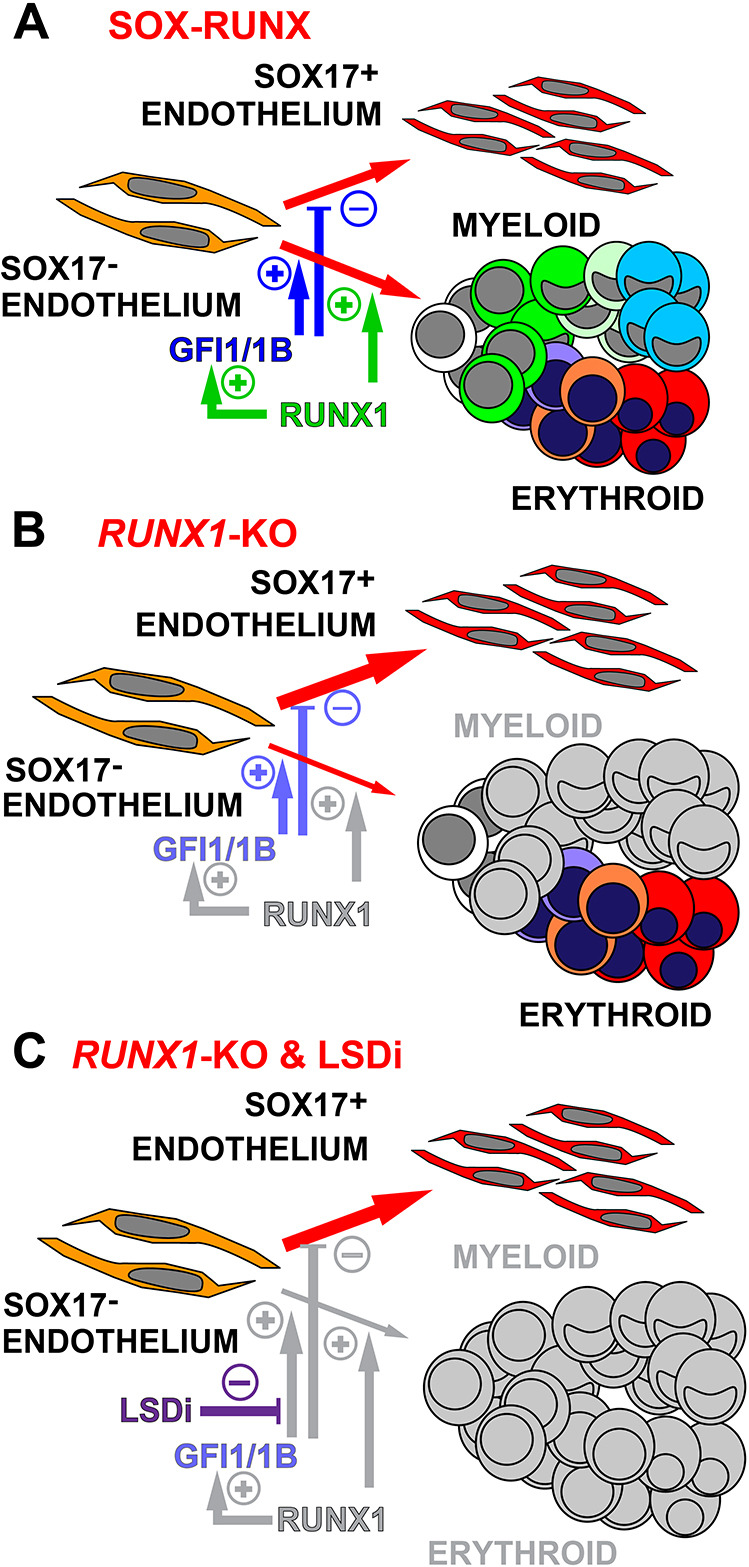
**Model of endothelial and haematopoietic differentiation in human blast colonies.** (A-C) Proposed differentiation pathway to blood and endothelium in blast colonies, illustrating the roles of *RUNX1*, *GFI1/1B* and LSD1 inhibition (LSDi). In the blood lineages, RUNX1C^+^ cells are shown in green whereas more mature myeloid cells that are RUNX1C^−^ are shown in blue. Erythroid cells are shown transitioning to red. Loss of gene expression or of blood lineages are shown in grey. Reduced levels of *GFI1/1B* in *RUNX1*-KO differentiations are shown as increased transparency of text. (A) Regulation of haematopoietic development within SOX-RUNX cultures, highlighting the postulated roles of *RUNX1* and *GFI1/1B* in regulating the pathways to blood and SOX17^+^ endothelium. Both haematopoietic and SOX17^+^ endothelial cells derive from the SOX17^−^ endothelial precursor, but we have not shown that both populations can arise from one cell. (B) In *RUNX1*-KO cells, RUNX1C^+^ cells and mature myeloid cells are absent, and *GFI1/1B* expression levels are reduced. (C) All blood cells are absent in cultures in which LSDi also blocks the functions of *GFI1/1*B.

Indeed, the d2 and d3 SOX17^−^ENDO cells expressed similar key haematopoietic genes to haemogenic endothelia in the mouse and human intra-embryonic AGM (Baron et al., 2018; Ng et al., 2016; Solaimani Kartalaei et al., 2015; Swiers et al., 2013), although *HOXA* expression was absent, as expected, from these extra-embryonic, yolk sac-like cells. Our study indicated that the d2 SOX17^−^ENDO population was the precursor of both CD34^+^CD43^+^ haematopoietic cells and a distinct SOX17-expressing endothelium, although we have not shown that one cell could give rise to both progeny (Fig. 7A). Our data are consistent with previous *in vitro* mouse ESC differentiation studies that found a transient population of Tie2^hi^c-Kit^+^CD41^−^ endothelial cells at d2 of blast colony differentiation that gave rise to CD41^+^ haematopoietic progeny (Lancrin et al., 2009). Indeed, RNA-seq analysis shows that the d2 SOX17^−^ENDO may be analogous to these mouse cells, in that they also expressed TEK (TIE2) and KIT, and were negative for ITGA2B (CD41) (Fig. 3E). Our work extends the mouse findings by demonstrating that this d2 SOX17^−^ENDO not only gave rise to blood cells, but also gave rise to a largely non haemogenic endothelium, now marked by the acquisition of *SOX17* and the loss of *RUNX1* expression.

Confirming the requirement for *RUNX1* in the haemogenic SOX17^−^ endothelium, we showed that deletion of *RUNX1* abrogated all haematopoiesis save for a single wave of extra-embryonic erythropoiesis (Fig. 7B). The results of these experiments can be taken to indicate that the blast colony-forming assay is not dominated by precursors of the first wave of extra-embryonic haematopoiesis, but predominantly reads out progenitors of a second, *RUNX1*-dependent wave of extra-embryonic haematopoiesis, perhaps analogous to a human EMP. This interpretation is supported by our ability to differentiate blast colonies to granulocytic cells, a lineage similarly generated from mouse yolk sac EMPs (Frame et al., 2013). Furthermore, our experiments also confirmed that all human extra embryonic, yolk sac-like macrophages were absolutely *RUNX1* dependent, consistent with reports in the mouse (Lacaud et al., 2002; Okuda et al., 1996; Wang et al., 1996).

We observed that loss of *RUNX1* led to reduced levels of *GFI1*/*1B*, as reported in the mouse (Lancrin et al., 2012; Thambyrajah et al., 2016b), but that expression was not completely lost (Fig. 4H and Fig. S5F). Exploring the role that residual GFI1/1B protein expression might play in the regulation of extra-embryonic erythropoiesis, we documented the complete suppression of blood formation by small molecule inhibition of the histone demethylase, LSD1. In the context of haematopoietic differentiation, LSD1 mediates the inhibitory effects of GFI1/1B on the endothelial program and *Lsd1* deficiency phenocopies the developmental block in endothelial-to-haematopoietic transition in *Gfi1*/*1b* null mouse embryos (Saleque et al., 2007; Thambyrajah et al., 2016a) (Fig. 7C). Consistent with this action, CD41^+^ cells in the yolk sacs of double knockout *Gfi1/Gfi1b* mouse embryos maintained endothelial gene expression, preventing their free migration and distribution into embryonic tissues outside the yolk sac. Although haematopoietic colonies formed *in vitro* from disaggregated yolk sacs, clonogenic cells were not found in the dorsal aorta and intra-arterial haematopoietic cluster formation was lost (Lancrin et al., 2012; Thambyrajah et al., 2016a). In our human pluripotent stem cell model, the complete loss of haematopoiesis in the presence of LSD1 inhibition suggests that the human extra-embryonic, yolk sac-like culture modelled in our hPSC *in vitro* differentiation may be more similar to the mouse AGM, rather than to yolk sac haemogenic endothelium. However, an important caveat remains that the effects of the chemical inhibitor may differ from the results that would be obtained from a double knockout of GFI1 and GFI1B genes.

Our data argue for a window of haematopoietic competence during blast colony differentiation in which expression of *GFI1/1B* reinforced by *RUNX1* drives the generation of blood and suppresses an endothelial program (Fig. 7). Such a model fits well with the narrow developmental window during which enforced *Runx1* expression in mouse embryonic endothelium is able to drive haematopoiesis (Yzaguirre et al., 2018). The factors that initiate *RUNX1* expression during this permissive stage are not known.

In summary, we have identified and characterised a population of SOX17*^−^* haemogenic endothelial cells that is the dominant source of blood and of SOX17^+^ endothelium in human extra-embryonic, yolk sac-like cultures. We have correlated *RUNX1* and *GFI1/1B* expression with increased haemogenic capacity, further identifying that *RUNX1* is required for human blast colony development. Finally, our studies also revealed the crucial role played by *GFI1*/*1B* in the emergence of the first erythroid cells and the absolute dependence of all other blood lineages on *RUNX1*.

## MATERIALS AND METHODS

### Ethics

Human pluripotent stem cell studies were approved by the Monash University (reference 2002/225MC) and The Royal Children's Hospital (reference 33001A) Human Research Ethics Committees.

### Generation and validation of targeted *SOX17*^mCHERRY/w^ *RUNX1C*^GFP/w^ and *SOX17*^mCHERRY/w^ *RUNX1*^−/−^ H9 hPSCs lines

The *SOX17*^mCHERRY/w^ H9*, SOX17*^mCHERRY/w^ *RUNX1C*^GFP/w^ (SOX-RUNX) H9 hPSC lines have been described previously (Loh et al., 2014; Ng et al., 2016). To generate the *SOX17*^mCHERRY/w^ *RUNX1*^−/**−**^ (*RUNX1*-KO) hPSC line, the CRISPR Design Tool (http://tools.genome-engineering.org) was used to design 18 nucleotide single-guide RNAs (sgRNAs) to target two sites within exon 4 of the *RUNX1* gene [5′ TGTCGCCGTCTGGTAGGA 3′ (CRISPR SITE 1) and 5′ GGTCGGTCTTCCTAGCTT 3′ (CRISPR SITE 2)] (Fig. S5A) (Ran et al., 2013). The corresponding 18 nucleotide sgDNAs were synthesised, phosphorylated and annealed to complementary sequences to make double-stranded (ds) DNA oligos. The oligos were modified to incorporate a 5′CACC and 3′CAAA overhang for BbsI site recognition within the pSpCas9-2A(BB)-GFP (PX458) vector, a gift from Feng Zhang (McGovern Institute, Cambridge, MA, USA; Addgene plasmid #48138), and 5′G-C base pair insertion to enable U6 transcription. The pSpCas9-2A-GFP vectors expressing the RUNX1 CRISPR 1 and 2 dsRNAs were electroporated into SOX-RUNX hPSCs. After 24-72 h, single GFP-positive cells were flow sorted and clonally expanded on pre-gelatinised 96-well plates. Clones were screened for the acquisition of a 457 bp deletion region of exon 4 of the *RUNX1* locus containing part of the DNA-binding domain using primers a and c (Fig. S5A,C). Positive clones were further selected for deletion verification by PCR using primers a and b, and Sanger sequenced using primers a and c. Three positive clones (11, 14 and 30) contained a complete deletion between CRISPR site 1 and CRISPR site 2. For all lines, surface markers of undifferentiated hPSCs were expressed and genomic integrity was confirmed by Illumina HumanCytoSNP-12 v2.1 array.

### Culture and differentiation of hPSCs

H9 hPSCs used in these studies were provided by the WiCell Research Institute. Cell lines were regularly tested to exclude mycoplasma contamination and confirm genomic integrity. Culture and enzymatic passaging of hPSCs lines was performed as previously reported (Ng et al., 2008b).

For the generation of blast colonies and for initial differentiation towards extra-embryonic, yolk sac-type haematopoietic cells, hPSCs were differentiated using the spin EB method in APEL medium (Ng et al., 2008a) supplemented for the first 2-3 days with 20 ng/ml recombinant human (rh) bone morphogenetic protein 4 (BMP4, R&D Systems), 30 ng/ml rh vascular endothelial growth factor (VEGF, PeproTech), 40 ng/ml rh stem cell factor (SCF, PeproTech) and 20 ng/ml rh activin A (R&D Systems).

In later differentiations towards extra-embryonic, yolk sac-type haematopoietic cells, hPSCs were differentiated as spin EBs in APEL medium supplemented for the first 4 days with 20 ng/ml rh BMP4, 25-30 ng/ml rh VEGF, 25-40 ng/ml rh SCF, 10-20 ng/ml rh activin A, 10 ng/ml rh fibroblast growth factor 2 (FGF2, PeproTech) and 0.5-1 µM CHIR99021 (CHIR, Tocris Biosciences). Where indicated, 1 µM GSK-LSD1 (lysine specific demethylase 1) inhibitor (Sigma Aldrich) was added to day2 EBs. For air-liquid interphase cultures, 30-60 EBs were transferred in 50 µl of growth factor-reduced (GFR)-Matrigel (BD Pharmingen) onto transwells at days 2, 3 and 4, and supplemented with the medium described above. Differentiation medium was changed after 4 days to APEL medium including 50 ng/ml rh SCF, 50 ng/ml rh VEGF, 20-25 ng/ml rh insulin like growth factor 2 (IGF2, PeproTech), 10 ng/ml rh FGF2 and, where indicated, 1 µM GSK-LSD1 inhibitor. At 6-7 days of differentiation, 20-30 EBs for submerged liquid culture were transferred onto gelatinised or GFR-Matrigel-treated (8 mg/ml) six-well plates in APEL medium supplemented with 50 ng/ml rh SCF, 50 ng/ml rh VEGF, 50 ng/ml rh interleukin (IL) 3 (PeproTech), 25 ng/ml rh thrombopoietin (TPO, PeproTech), 25 ng/ml rh FLT3 receptor ligand (FLT3L, PeproTech), 10 ng/ml rh FGF2 and 20-30 ng/ml rh erythropoietin (EPO, PeproTech). Air-liquid interphase EBs were cultured in APEL medium containing 100 ng/ml rh SCF, 100 ng/ml rh TPO, 25 ng/ml rh VEGF, 25 ng/ml rh FLT3L, 25 ng/ml rh interleukin (IL) 6 (PeproTech) and 10 ng/ml rh IGF2. Medium was changed every 5-7 days for submerged liquid cultures or every 1-2 days for air-liquid interphase cultures.

For intra-embryonic haematopoietic differentiation, cells were differentiated using the spin EB method in APEL medium supplemented for the first 4 days with 20 ng/ml rh BMP4, 25 ng/ml rh VEGF, 25 ng/ml rh SCF and 10 ng/ml rh activin A, 10 ng/ml rh FGF2 and 0.5-1 µM CHIR. Between days 2 and 4, 3 µM CHIR99021 and 3-4 µM SB431542 (SB, Cayman Chemicals) were added to pattern mesoderm. Medium was changed after 4 days to APEL medium supplemented with 20 ng/ml rh BMP4, 50 ng/ml rh SCF, 50 ng/ml rh VEGF, 10-20 ng/ml rh IGF2 and 10 ng/ml rh FGF2. After 6-8 days of differentiation, EBs were transferred onto GFR-Matrigel-treated six-well plates or air-liquid interphase transwells at 20-60 EBs/well in APEL medium supplemented with 100 ng/ml rh SCF, 25-100 ng/ml rh FLT3 L, 25-50 ng/ml rh VEGF, 50-100 ng/ml rh TPO, 25 ng/ml rh IL6, 10 ng/ml rh FGF2 and 10 ng/ml rh BMP4. Medium was changed every 3-5 days thereafter. For analysis, EBs, liquid, air-liquid interphase and methylcellulose cultures were harvested and dissociated into single cell suspension using TrypLE Select (Invitrogen) or Collagenases Type I and IV (Worthington) and passed through a 21-23-gauge needle and 40 µm cell strainer.

### Colony-forming assays and culture of sorted cells

Blast colony-forming cells (BL-CFCs) were identified by culturing 3×10^3^-1×10^4^ dissociated cells, or 3×10^4^ cells for high density BL-CFC assays, from day 2 or 3 EBs in a formulation designated MC-APEL (1% methylcellulose in APEL medium) or serum-free MethoCult (StemCell Technologies) supplemented with 100 ng/ml rh SCF, 50 ng/ml rh VEGF, 50 ng/ml rh IL-3, 50 ng/ml rh IL-6, 50 ng/ml rh TPO, 20 ng/ml rh BMP4, 10 ng/ml rh FGF2 and 2 μg/ml rh EPO. Where indicated, methylcellulose cultures were also supplemented with one of the following inhibitors or cytokines: 1 mM GSK-LSD1, 50 ng/ml rh macrophage colony-stimulating factor (M-CSF, PeproTech), 50 ng/ml rh granulocyte colony-stimulating factor (G-CSF, R&D Systems) and 50 ng/ml rh granulocyte-macrophage colony-stimulating factor (GM-CSF, R&D Systems). Colony formation was scored between 7 and 10 days of differentiation. After 3 to 7 days of blast colony formation, unsorted or cell sorted populations were cultured on GFR-Matrigel-coated plates in APEL medium supplemented with 100 ng/ml rh SCF, 50 ng/ml rh VEGF, 50 ng/ml rh IL3, 50 ng/ml rh IL6, 50 ng/ml rh TPO, 20 ng/ml rh BMP4, 10 ng/ml rh FGF2, 20 ng/ml rh erythropoietin and where indicated 50 ng/ml rh M-CSF, 50 ng/ml rh G-CSF and 50 ng/ml rh GM-CSF for 3 to 7 days. Later appearing haematopoietic colonies were identified by culturing 1-3×10^4^ dissociated cells from day 7-25 cultures in a formulation designated MC-APEL or serum-free MethoCult supplemented with 100 ng/ml rh SCF, 50 ng/ml rh VEGF, 50 ng/ml rh IL-3, 50 ng/ml rh IL-6, 50 ng/ml rh TPO, 50 ng/ml rh G-CSF, 10 ng/ml rh FGF2, 20 ng/ml rh erythropoietin and 10 µg/ml human low-density lipoproteins (LDL, Stem Cell Technologies). Colony formation was scored between 10 and 15 days of differentiation.

### Limit dilution estimation of frequency of haematopoietic precursor frequency

To determine the clonal frequency of haematopoietic precursors, day 2+2 and day 2+3 blast colonies were flow sorted on the basis of CD34, CD43 and SOX17 expression, and cells deposited by flow cytometry at 1, 3, 10, 30, 100 and 300 cells per well into GFR-matrigel coated 96-well plates. After 5-10 days of culture in APEL medium supplemented with 100 ng/ml rh SCF, 50 ng/ml rh VEGF, 50 ng/ml rh IL-3, 50 ng/ml rh IL6, 50 ng/ml rh TPO, 20 ng/ml rh BMP4, 10 ng/ml rh FGF2 and 20 ng/ml rh erythropoietin, wells were scored by microscopy for the presence of hematopoietic clusters of greater than 30 cells. The frequency of colony-forming cells was estimated using Poisson statistics.

### Endothelial network assay and time lapse imaging

GFR-Matrigel was solidified at 37°C for 30 min in wells of a 48-well plate. For the experiments shown in Fig. 1, 5×10^4^, day 2+3 flow-sorted SOX17^+^ENDO or SOX17^−^ENDO cells were seeded onto polymerised GFR-Matrigel in APEL medium supplemented with 50 ng/ml rh VEGF, 10 ng/ml rh FGF2, 5 ng/ml rh epidermal growth factor (EGF, PeproTech) and 10^−3^ hydrocortisone (StemCell Technologies) and incubated for 24 h. After 48 h, APEL medium was supplemented with 100 ng/ml rh SCF. For the experiments shown in Fig. 2, Fig. S2 and Movie 1, 1.2×10^5^, day 2+1.75 flow-sorted SOX17^−^ENDO (SOX17^−^CD34^+^CD43^−^CD73^−^) cells were seeded onto polymerised GFR-Matrigel in APEL medium supplemented with 50 ng/ml rh VEGF, 100 ng/ml rh SCF, 10 ng/ml rh FGF2, 5 ng/ml rh EGF and 10^−3^ hydrocortisone. For the experiment in Fig. S2, cultures were incubated in a 5% CO_2_ incubator at 37°C and wells were imaged at 24 h and 48 h after the endothelial network assay was set up. For the experiment in Fig. 2, cultures were incubated in a 5% CO_2_ incubator at 37°C for 6 h and then placed in an environmentally controlled (37°C, 5% CO_2_ in humidified air) chamber fitted to a Zeiss LSM 780 laser scanning confocal microscope for time-lapse imaging.

### Flow cytometry and cell sorting

Antibodies directed against the following cell surface antigens [fluorochrome, manufacturer, catalogue number, clone (where known), dilution] were used to stain dissociated cells for flow cytometric analysis: CD14 [phycoerythrin(pe)-cy7, BioLegend 301814, clone M5E2, 1:50], CD31 [allophycocyanin (apc), BioLegend 303115, clone WM59, 1:50; brilliant violet (bv)-421, BioLegend 303123, clone WM59, 1:30], CD34 (pe-cy7, BioLegend 43515, clone 581, 1:100), CD43 (apc, BioLegend 343206, clone 10G7, 1:50; bv-421, BD Pharmingen 562916, clone 1G10, 1:30), CD45 (bv-421, BioLegend 304032, clone H130, 1:30), CD73 (apc, BioLegend 344006, clone AD2, 1:50), glycophorin A (GYPA) [apc, BD Pharmingen 551336, clone GA-R2(HIR2), 1:2000], platelet-derived growth factor receptor alpha (PDGFRα) (BD Pharmingen 556001, clone aR1, 1:100), vascular endothelial growth factor receptor 2 (VEGFR2/KDR) [Alexa fluor (af)-647, BioLegend 338909, clone HKDR-1, 1:10] and TIE2/TEK (BD Pharmingen 557039, clone 33, 1:100). PDGFRα- and TIE2-unconjugated antibodies were detected with secondary antibodies conjugated with apc (BD Pharmingen 550826, 1:100; BioLegend 405308, clone Poly4053, 1:100) or pe-cy7 (BioLegend 405315, clone Poly4053, 1:100). Flow cytometric analysis was performed using a BD LSR Fortessa analyser. Flow sorting used a BD Biosciences Influx or BD Biosciences FACSAria Fusion cell sorter.

Samples were gated using FSC-A and FSC-H to exclude doublets. In some cases, FSC-W, SSC-A and SSC-H were also used. FSC and propidium iodide exclusion were used to select live cells. Positive gates for markers of interest were determined by comparing stained samples with those in which the antibodies were not added. Frequently, this could also be corroborated by gating on samples in which the marker under evaluation was not expressed. There are numerous flow cytometry plots in this article. The example chosen to illustrate gating strategy is one of the experiments from which samples were sorted for RNA-sequencing analysis. This is shown in Fig. S1A.

### Gene expression analysis

Total RNA was isolated from hPSCs using the RNA Isolate II Mini or Micro Kits (Bioline) or RNeasy Kit (Qiagen) as specified by the manufacturer. cDNA was reverse transcribed via random hexamer priming and Tetro cDNA synthesis (Bioline) or Superscript III (Invitrogen) kits in accordance with the manufacturers' instructions. TaqMan gene expression probes (Applied Biosystems) and Bioline reagents were used for quantitative real-time PCR using *GAPDH* as the reference gene to normalise the data.

TaqMan assays directed towards the following target sequences were used to detect gene expression: CD34 (Hs00156373_m1), CSFR1 (Hs00234622_m1), EPX (Hs00946094_m1), GAPDH (Hs99999905_m1), GATA1 (Hs00231112_m1), GATA2 (Hs00231119_m1), GFI1 (Hs00382207_m1), GFI1B (Hs01062469_m1), HAEMOGLOBIN-ALPHA (Hs00361191_g1), HAEMOGLOBIN-BETA (Hs00747223_g1), HAEMOGLOBIN-EPSILON (Hs00362215_g1), HAEMOGLOBIN-GAMMA (Hs00361131_g1), HAEMOGLOBIN-ZETA (Hs00923579_m1), KLF1 (Hs00610592_m1), mCHERRY (custom design by ThermoFisher), RUNX1 (Hs00231079_m1), RUNX1C (Hs01021967_m1), SOX17 (Hs00751752_s1), SPI1 (Hs00231368_m1) and SPN (CD43) (Hs01872322_s1).

### Transcriptional profiling using RNA-sequencing

In the first series of experiments (series 1), differentiated SOX-RUNX (*SOX17*^mCHERRY/w^*RUNX1C*^GFP/w^) cell cultures were harvested from EBs at day 2 and from methylcellulose cultures at day 2+2 and day 2+3 of culture, and flow sorted based on their expression of PDGFRα, CD34, CD43 and mCHERRY using a BD Biosciences Influx or BD Biosciences FACSAria Fusion cell sorter. In a second series of experiments (series 2), cultures of SOX-RUNX and *RUNX1*-KO (*SOX17*^mCHERRY/w^*RUNX1*^−/−^) cells differentiated for 4 or 6 days in the presence or absence of 1 µM GSK-LSD1 inhibitor were harvested without additional sorting. Total RNA was purified from samples (RNA Isolate II Micro Kit, Bioline) and RNA concentration was determined using a Nanodrop 2000 analyser (Thermo Scientific). Total RNA from the differentiated fractions was sequenced at the Australian Genome Research Facility (series 1) or the Murdoch Children's Research Institute (series 2). In total, 16 samples from the series 1 (two to four independent experiments for each sample) and 24 samples from series 2 (three independent experiments for each sample) were sequenced. The STAR aligner (v2.4.0h1 or v2.5.2a) in 2 pass mode was used to map single end 100 bp reads to the human reference genome (hg38) for series 1, and 75 bp paired end reads to the human reference genome (hg39) for series 2 (Dobin et al., 2013). Uniquely mapped reads were summarised using featureCounts (v1.4.6) using Gencode Release 19 comprehensive annotation (Liao et al., 2014). Genes lowly expressed were excluded (fewer than 10 counts per million in fewer than two samples in series 1, and fewer than 1 count per million in fewer than three samples in series 2). The data was TMM normalised, voom transformed and differential gene expression was assessed using moderated *t*-tests from the R Bioconductor limma package (Ritchie et al., 2015; Robinson and Oshlack, 2010). Genes that had a false-discovery rate of less than 5% were classed as significantly differentially expressed for the various comparisons of interest. Gene ontology analysis was performed using the ToppGene Suite (Chen et al., 2009a).

### Confocal microscopy and image processing

Confocal images (Fig. 1E,J-M; Fig. 2C; Fig. 4A-D; Fig. 5G; Fig. 6F-H; Fig. S2B,C and Movie 1) were acquired as single optical sections on a Zeiss LSM 780 laser scanning confocal microscope running Zeiss Black software. Overlay images were created in Adobe Photoshop 2020 release 21.1.0 and figures prepared in Adobe Illustrator 2020 release 24.1. The only image manipulations performed were adjustments of brightness and contrast. The time-lapse images, counting of SOX17-mCHERRY fluorescent cells and counting the bright-field cells in the endothelial tube assay for Fig. 2C and Movie 1 were performed using Fiji (ImageJ) open source image processing software v2.0.0-rc-69/1.52p (https://imagej.nih.gov/).

### Statistical analysis

Experiments were analysed using GraphPad Prism versions 58 and Microsoft Excel for Mac version 13.36. Tests for statistical significance are listed with each experiment and included two-sided Student's *t*-test for paired analyses, multiple *t*-tests with Sidak-Bonferroni post-hoc test, or ANOVA for experiments with multiple comparisons of one or more grouped variables, accompanied by the post-hoc tests (Dunnett's, Tukey's or Holmes-Sidak) indicated as appropriate by the software. No statistical method was used to predetermine sample size.

## Supplementary Material

Supplementary information

Reviewer comments
