# Supplementary material for: Human yolk sac-like haematopoiesis generates RUNX1-, GFI1- and/or GFI1B-dependent blood and SOX17-positive endothelium
Source: Development. 2020 Oct 29;147(20):dev193037. doi: 10.1242/dev.193037 (PMC7648599; doi:10.1242/dev.193037)
Supplement: Supplementary information [file develop-147-193037-s1.pdf]

SUPPLEMENTARY TABLES

Table S1. Haematopoietic precursor frequency in blast colony sorted populations.

Click here to Download Table S1

Table S2. RNA-Sequencing analysis of sorted d2 mesoderm and haematopoietic blast colony populations from d2 cultures following d2 (d2+2) or d3 (d2+3) in methylcellulose.

Click here to Download Table S2

Table S3. Gene ontology terms for mesoderm and haematopoietic blast colony populations.

Click here to Download Table S3

Table S4. Differentially expressed genes between sorted d2 mesoderm and haematopoietic blast colony populations from d2 cultures after d2 (d2+2) or d3 (d2+3) in methylcellulose.

Click here to Download Table S4

Table S5. RNA-Sequencing analysis of control and LSD1 inhibitor treated d4 and d6 SOX-RUNX and *RUNX1*-KO cultures.

Click here to Download Table S5

Table S6. Differentially expressed genes between control and LSD1 inhibitor treated d4 and d6 SOX-RUNX cultures.

Click here to Download Table S6

Table S7. Differentially expressed genes between d6 SOX-RUNX and d6 *RUNX1*-KO cultures.

Click here to Download Table S7

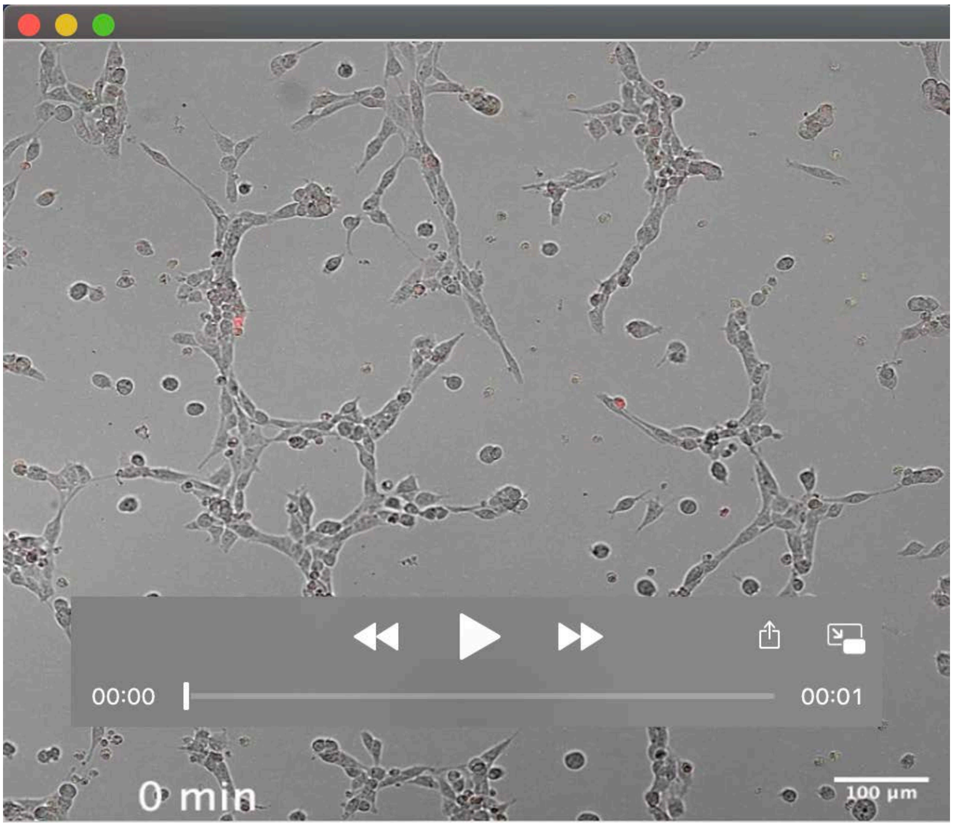

Movie 1. Time lapse series of endothelial network assay. Series of images taken at 10 min intervals from 0 to 3920 min (65 hours) as indicated on each image. Images run at 10fps. The first faintly red fluorescent SOX17^{+} cells are seen from 360 min (6 hours) and are obvious from 720 min (12 hours) onwards. Blood cells appear from 2160 min (36 hours). Scale bar, 100µm. Related to **Fig. 2.**

Development • Supplementary information

SUPPLEMENTARY FIGURES

Bruveris Figure S1

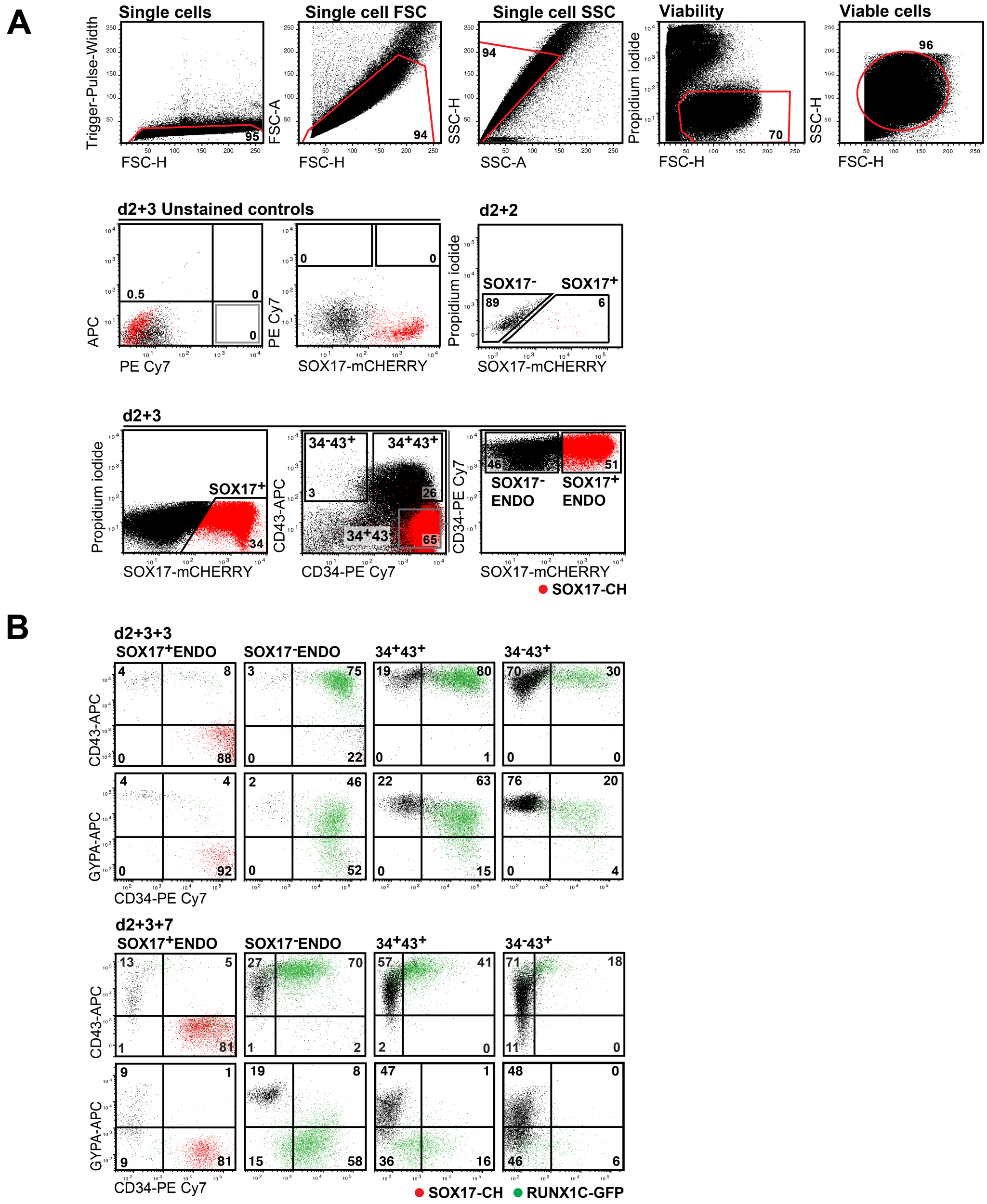

Figure S1. Haematopoietic development from SOX-RUNX blast colonies. (A) Example of flow cytometry gating strategy for data shown in **Fig. 1H**. Top panel of plots describes single and viable cell isolation process, lower panels illustrate unstained controls, SOX17$^+$ and SOX17$^-$ cell separation and subsequent surface marker population isolation. **(B)** Flow cytometric analysis of CD34, CD43, GYPA expression in sorted fractions of d2 blast colonies after 3 days in methylcellulose (d2+3) (see **Fig. 1H)** following three (d2+3+3) and seven (d2+3+7) days of re-culture. By 7d, d3 SOX17$^+$ENDO fractions remain primarily as endothelium with a few GYPA$^+$CD34$^-$ erythroid cells, while the d3 SOX17$^-$ENDO fractions contain RUNX1$^+$CD34$^+$ myeloid and GYPA$^+$CD34$^-$ erythroid cells. The greatest proportion of erythroid cells arises from the first blood cells to emerge, 34$^-$43$^+$ (n=3 experiments). Mean±SEM provided in text for selected markers. Data for d2+3+5 shown in **Fig. 1I**.

Bruveris Figure S2

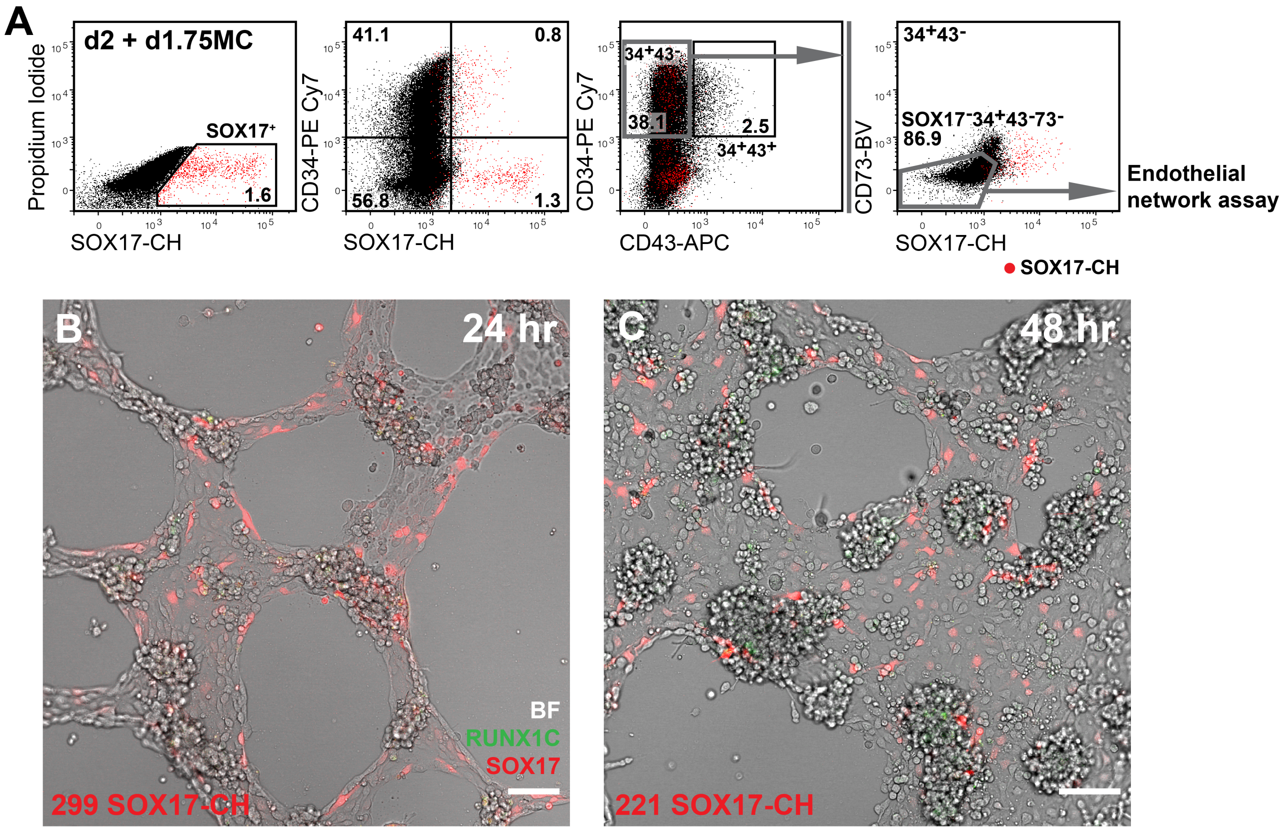

Figure S2. Endothelial network assay showing generation of SOX17-positive from SOX17-negative endothelium. (A) Gating strategy outlining the sorting of SOX17$^-$ENDO cells from methylcellulose at d2+d1.75. The two left-hand panels show the expression of SOX17 and of CD34 at this time point. The right-hand panels show the sequential gating strategy used to sort the SOX17$^-$ ENDO (SOX17$^-$CD34$^+$CD43$^-$CD73$^-$) endothelial cells that were seeded into the endothelial network assay. This is an independent experiment to that shown in **Fig. 2**. **(B, C)** Images taken from the endothelial network assay at (B) 24 hr and (C) 48 hr time points. SOX17$^+$ and SOX17$^-$ adherent cells are seen as well as developing clusters of haematopoietic cells. This experiment shows more rapid differentiation than the time lapse experiment in **Fig. 2**, suggesting that the environmental control achieved in a sealed incubator (this experiment) is superior to differentiation in the controlled climate (37°C, 5% CO_2 in humidified air) chamber used for the time lapse series. The number of SOX17$^+$ endothelial cells in each image is indicated. The number of tightly packed haematopoietic cells precluded accurate assessment of the total cell number. Scale bar, 100µm.

Bruveris Figure S3

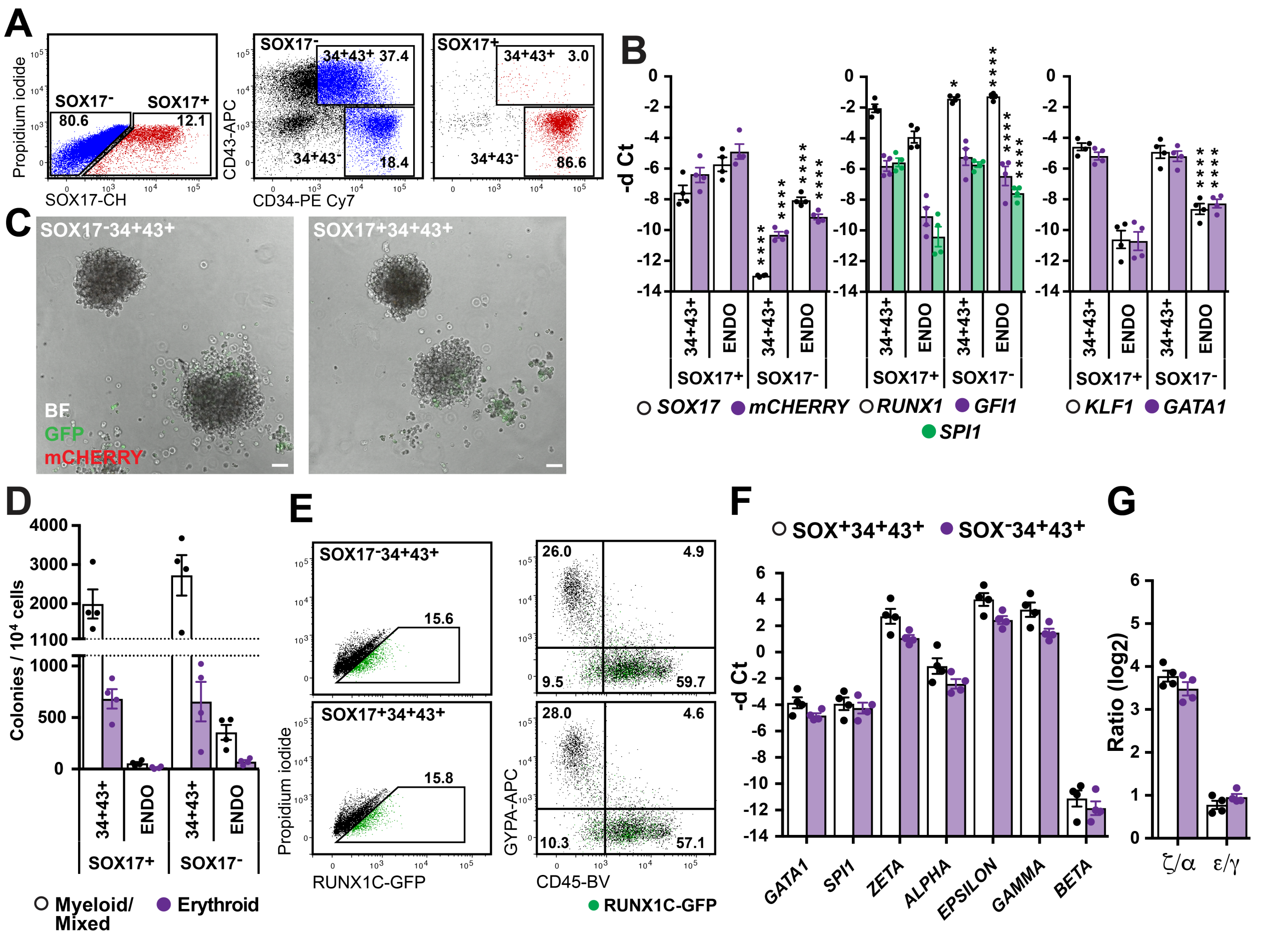

Figure S3. (A) Sorting strategy for d2+3 SOX17^{+} and SOX17^{-} blood (34^{+}43^{+}) and endothelial (ENDO [34^{+}43^{-}]) fractions (n=4 experiments). **(B)** PCR analysis of d2+3 sorted fractions from (A) correlating SOX17-mCHERRY reporter expression with *SOX17*, and expression of blood genes (*RUNX1, GFI1, SPI1, KLF1, GATA1*) in the SOX17^{+} compared to SOX17^{-} populations (mean±SEM, n=4 experiments). * *P*=0.048; **** *P*<0.0001, 2-way ANOVA, Tukey's multiple comparisons test. **(C)** SOX17^{+}34^{+}43^{+} and SOX17^{-}34^{+}43^{+} sorted fractions after d9 of methylcellulose re-culture (d2+3+9) displayed similar haematopoietic colony morphology. Scale bar, 50μm (n=4 experiments). **(D)** Clonogenic frequency was similar between the SOX17^{-} and SOX17^{+} blood (34^{+}43^{+}) fractions (mean±SEM, n=4 experiments). **(E)** Analysis of sorted fractions after d11 of methylcellulose re-culture (d2+3+11) illustrated similar RUNX1C, CD45 and GYPA expression. Plots are representative of four experiments. **(F)** Relative gene expression (shown as negative delta (-d) Ct) analysis of haematopoietic (*GATA1*, *SPI1*) and globin genes (*ZETA*, *ALPHA*, *EPSILON*, *GAMMA*, *BETA*) from d2+3+11 methylcellulose colonies in (D) (mean±SEM, n=4 experiments). **(G)** Ratio of embryonic globin genes showing a similar ratio of ζ/α and ε/γ indicating a predominance of primitive globins (mean±SEM, n=4 experiments).

Bruveris Figure S4

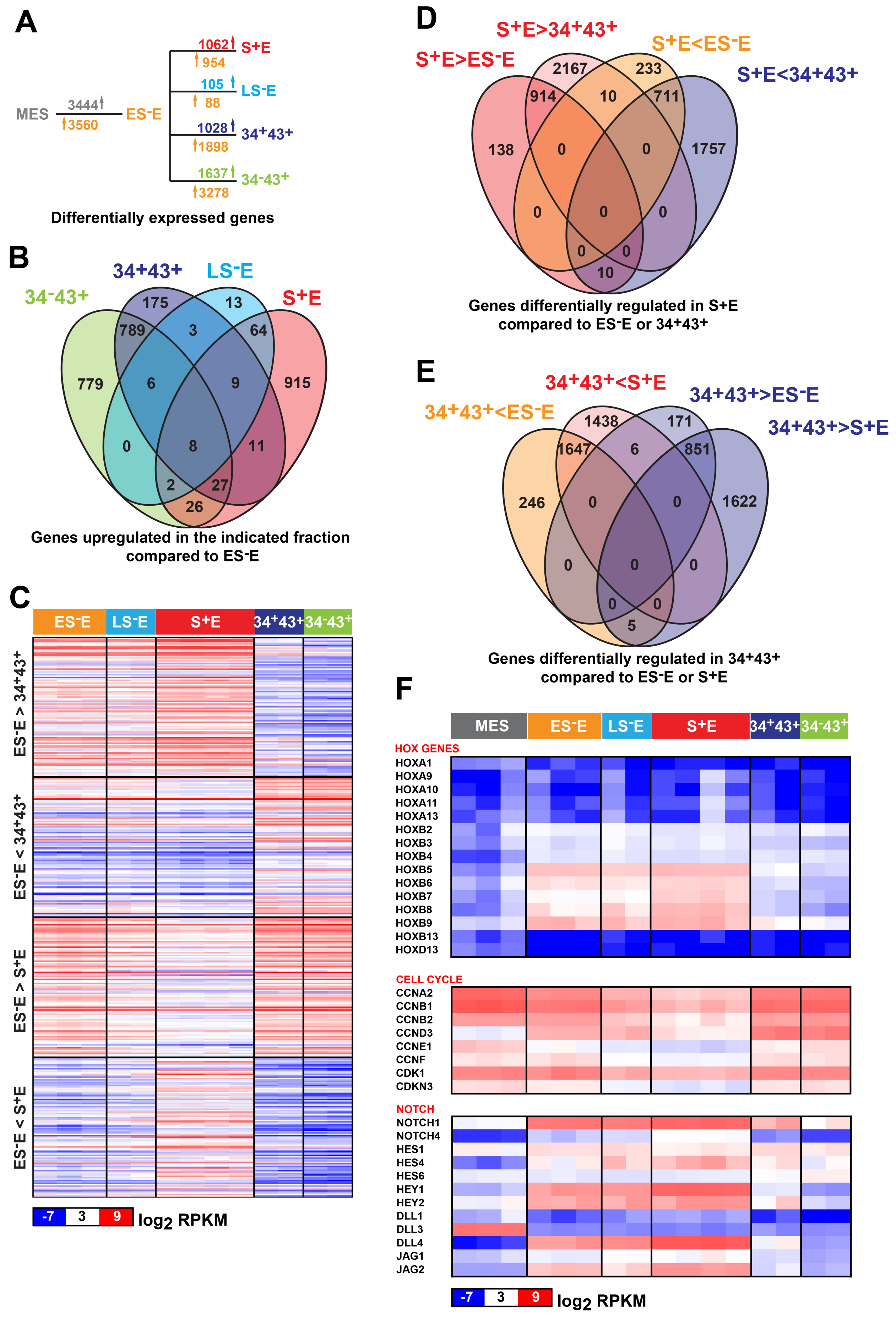

Development • Supplementary information

Figure S4. SOX17-negative endothelium represents a divergent point between endothelial and blood commitment. (A) Summary of differentially expressed genes between indicated mesoderm (MES, grey), d2 $SOX17^{-}$ENDO ($d2S^{-}E$, orange), d3 $SOX17^{+}$ENDO ($d3S^{+}E$, red), d3 $SOX17^{-}$ENDO ($d3S^{-}E$, cyan) and haematopoietic ($34^{+}43^{+}$, dark blue; $34^{+}43^{-}$, green) sorted fractions. See **Fig. 3A** for flow-sorting strategy. Genes are listed in **Supplementary Table 4**. **(B)** Venn diagram showing up regulated genes comparing $d2S^{-}E$ to indicated sorted fractions. Genes are listed in **Supplementary Table 4. (C)** RNA-Seq heatmap of sorted endothelial and haematopoietic populations showing differentially expressed genes compared to the $d2S^{-}E$ population. In the upper two panels, genes up or down regulated in $34^{+}43^{+}$ blood cells tend to be reciprocally expressed in all endothelial populations. In the lower two panels, genes up or down regulated in $d3S^{+}E$ or were reciprocally expressed in haematopoietic fractions. Thus, the $d2S^{-}E$ population includes genes in common with both the $d3S^{+}E$ and $34^{+}43^{+}$ populations, Scale, log_2 RPKM. Genes are listed in **Supplementary Table 4. (D, E)** Venn diagrams illustrating differentially regulated genes in $d3S^{+}E$ compared to $d2S^{-}E$ or $34^{+}43^{+}$ **(D)** and $34^{+}43^{+}$ compared to $d2S^{-}E$ or $d3S^{+}E$ **(E)**. Genes are listed in **Supplementary Table 4. (F)** RNA-Seq heatmap of sorted fractions showing gene expression of *HOX*, cell cycle and *NOTCH* pathway genes. No reads were detected for members of the *HOX* clusters that are not shown. Scale, log_2 RPKM.

Development • Supplementary information

Bruveris Figure S5

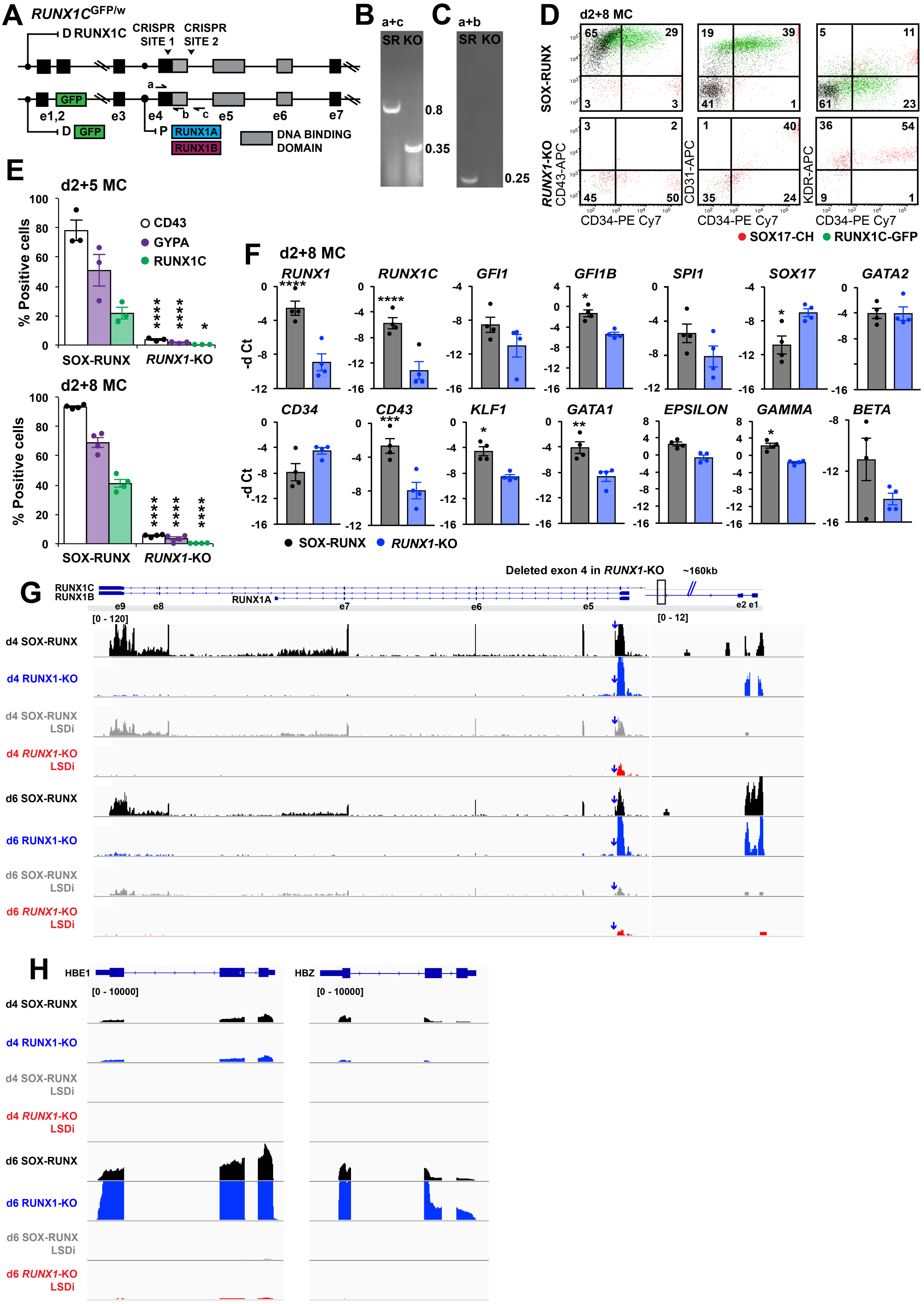

Figure S5. Impaired haematopoiesis in *RUNX1*-KO cells (A) Schematic representation of the *RUNX1C* locus showing wild-type and GFP targeted alleles, and sites for CRISPR deletion to create the *RUNX1*-KO cell line. Exons, e1 to e7, screening primers (a-c), proximal (P) and distal (D) promoters and DNA binding domain (grey) are indicated. **(B-C)** PCR screening confirmed the homozygous CRISPR deleted region in the *RUNX1*-KO (KO), compared to SOX-RUNX (SR)**.** PCR product lengths are indicated and screening primer locations are shown in (A). **(D)** Flow cytometry plots of d2 cells after eight days of methylcellulose (d2+8MC) culture of SOX-RUNX and *RUNX1*-KO cells illustrate the absence of $CD43^+$ blood cells in *RUNX1*-KO (n=4 experiments). See also **Fig. 4F,G**. **(E)** Summary of flow cytometry analysis, comparing haematopoietic gene expression in SOX-RUNX and *RUNX1*-KO cells after 5 (d2+5) and 8 (d2+8) days in methylcellulose (d2+5, mean±SEM, n=3; d2+8, mean±SEM, n=4 experiments). * P=0.017, **** P<0.0001 compared to SOX-RUNX, 2-way ANOVA, Holm Sidak's multiple comparisons test. **(F)** Relative gene expression, displayed as negative delta Ct (-d Ct), for selected haematopoietic genes in d2+8 methylcellulose *RUNX1*-KO cultures compared to SOX-RUNX controls, illustrating down regulation of haematopoietic gene expression in *RUNX1*-KO cultures (mean±SEM, n=4 experiments). * P<0.05, ** P<0.001, *** P<0.0002, **** P<0.0001 compared to SOX-RUNX, 2-way ANOVA, Holm Sidak's multiple comparisons test. See also **Fig. 4H. (G)** Integrative Genomics Viewer window showing RNA-Seq reads over the *RUNX1* locus in d4 and d6 SOX-RUNX and *RUNX1*-KO lines with and without treatment with the LSD1 inhibitor (LSDi). Note the absence of transcripts down stream of the deleted exon 4 in the *RUNX1*-KO lanes but retention of normal levels of transcripts up stream of the deletion. It is clear that LSD1 inhibition reduces *RUNX1* transcripts from both the distal and proximal promoters. The CRISPR deleted region in exon 4 (e4) is shown and marked with an arrow in each lane. (n=3 experiments). **(H)** Integrative Genomics Viewer window showing RNA-Seq reads over the *HBE1* and *HBZ* loci. The levels of embryonic globin transcripts are increased in the *RUNX1*-KO lanes compared to SOX-RUNX lanes, but transcripts are extinguished following the addition of LSD1 inhibitor.

Development • Supplementary information

Bruveris Figure S6

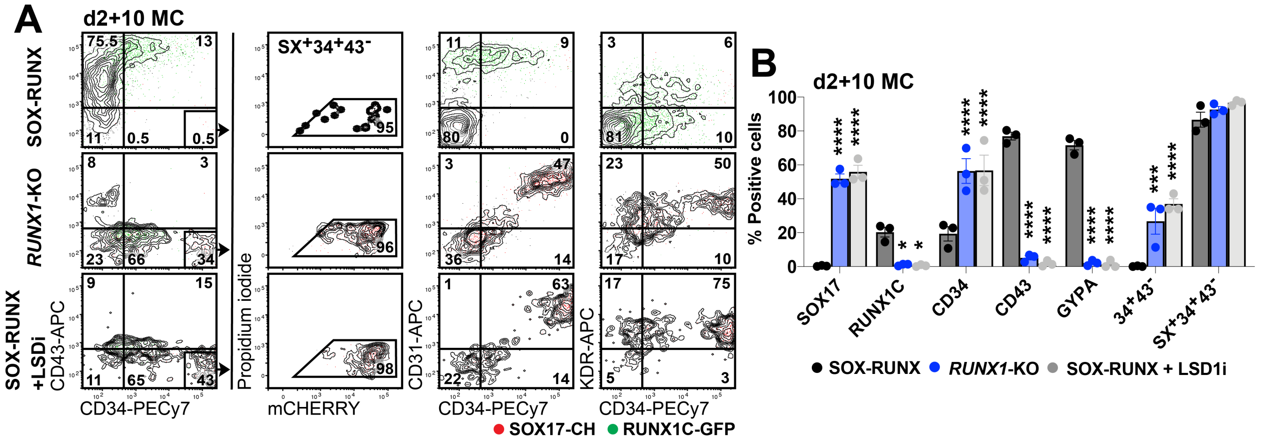

C

Category	ID	Name	p-value	q-value Bonferroni
**d6 LO in LSDi**				
GO: Biological Process	GO:0015671	oxygen transport	1.36E-11	7.38E-08
GO: Cellular Component	GO:0005833	hemoglobin complex	5.41E-13	5.41E-13
**d6 HI in LSDi**				
GO: Biological Process	GO:0006811	ion transport	9.96E-10	6.98E-06
Drug	ctd:C496932*	panobinostat	5.09E-38	1.68E-33

* Comparative Toxicogenomics Database

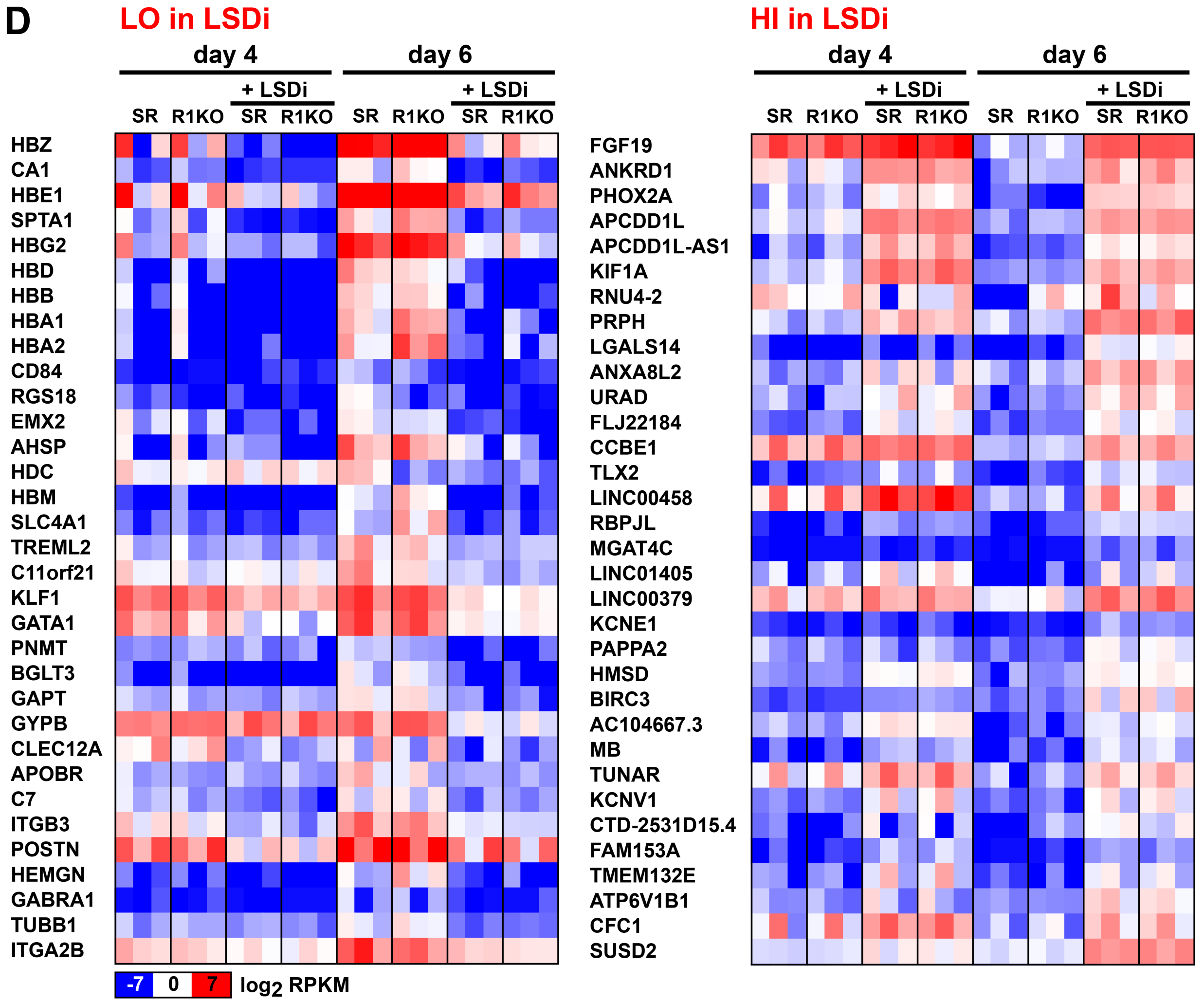

Development • Supplementary information

Figure S6. **RNA-Seq of LSD1 inhibitor-treated cultures. (A)** Flow cytometry plots of day 10 methylcellulose cultures (d2+10MC) of SOX-RUNX, *RUNX1*-KO and LSD1 inhibitor treated SOX-RUNX cells, showing that similar endothelium is generated by both the SOX-RUNX and *RUNX1*-KO cell lines. The proportion of $34^{+}43^{-}$ endothelium expressing SOX17 ($SX^{+}34^{+}43^{-}$) is highlighted. Plots are representative of three experiments. **(B)** Summary of flow cytometry analysis of the SOX-RUNX, *RUNX1*-KO and SOX-RUNX cells supplemented with LSD1 inhibitor shown in (A) (mean±SEM, n=3 experiments). * P<0.05, *** P<0.0002, **** P<0.0001 compared to SOX-RUNX, 2-way ANOVA, Holm Sidak's multiple comparisons test. **(C)** Gene ontology (GO) terms related to genes downregulated (LO) in RNA-Seq data from d6 LSD1 inhibitor (LSDi) treated cultures reflected the loss of blood cell differentiation, oxygen transport and hemoglobin complex. GO terms related to genes with higher (HI) expression in the same cultures related to ion transport and, more significantly, genes up regulated by the HDAC inhibitor panobinostat. **(D)** Heatmaps of RNA-Seq data from d4 and d6 SOX-RUNX (SR) and *RUNX1*-KO (R1KO) ± LSD1 inhibitor (LSDi) of the most differentially expressed genes down regulated (LO) or up regulated (HI) in LSDi supplemented compared to un-supplemented cultures. Scale, $\log_2$ RPKM. See also **Fig. 6M**.

Development • Supplementary information

Bruveris Figure S7

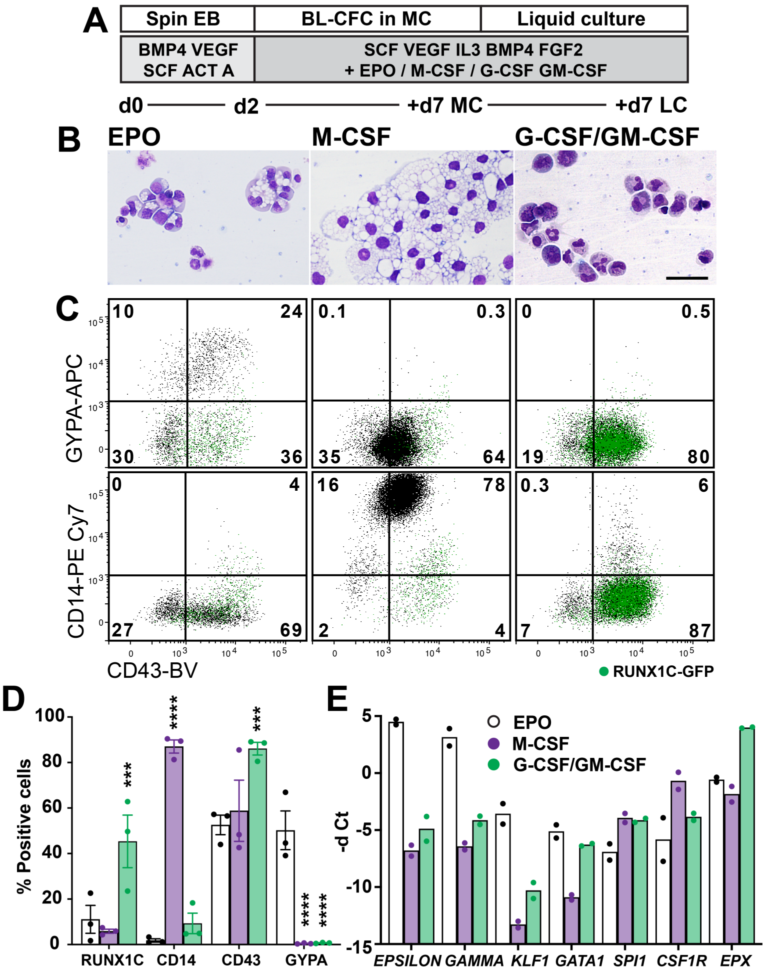

Figure S7. Human blast colonies generate *RUNX1*-dependent yolk sac-like cells. (A) Differentiation protocol for lineage specification. BL-CFC, blast colony forming cell; MC, methylcellulose; LC, liquid culture. **(B)** Images of May-Grünwald-Giemsa stained cytocentrifuge preparations of d2+7+7 cultures illustrating appearance of erythroid cells in EPO cultures, macrophages in M-CSF supplemented cultures and granulocytes exclusively in G-CSF/GM-CSF treated cultures. Images are representative of three experiments. Scale bar, 100μm. **(C)** Flow cytometric profiles of cultures showing expression of GYPA, CD14 and CD43 to distinguish between erythroid, macrophage and granulocytic lineages **(D)** Quantification of flow cytometric analysis illustrating the predominance of RUNX1C expression in the G-CSF/GM-CSF treated cultures, CD14 in the M-CSF supplemented cultures and GYPA in the EPO treated cultures (mean±SEM n=3 experiments). *** *P*<0.0002, **** *P*<0.0001 compared to EPO supplemented cultures, 2-way ANOVA, Holm Sidak's multiple comparisons test. **(E)** Relative gene expression (shown as negative delta (-d) Ct) analysis of d2+7+7 cultures demonstrating the expression of EPSILON and GAMMA globin, *KLF1* and *GATA1* in EPO supplemented cultures, *CSF1R* and *SPI1* in M-CSF supplemented cultures and *EPX*, *SPI1* and *GATA1* in G-CSF/GM CSF supplemented cultures, consistent with the development of erythroid, macrophage and granulocytic lineages respectively (mean of n=2 experiments). Data presented in panels B and C are from the same experiment.

Development • Supplementary information
