# Supplementary material for: Human yolk sac-like haematopoiesis generates RUNX1-, GFI1- and/or GFI1B-dependent blood and SOX17-positive endothelium
Source: Development. 2020 Oct 29;147(20):dev193037. doi: 10.1242/dev.193037 (PMC7648599; doi:10.1242/dev.193037)
Supplement: Reviewer comments [file develop-147-193037_review_history.pdf]

Human yolk sac-like haematopoiesis generates *RUNX1*-, *GFI1*- and/or *GFI1B*-dependent blood and *SOX17*-positive endothelium

Freya F. Bruveris, Elizabeth S. Ng, Ana Rita Leuitoguinho, Ali Motazedian, Katerina Vlahos, Koula Sourris, Robyn Mayberry, Penelope McDonald, Lisa Azzola, Nadia M. Davidson, Alicia Oshlack, Edouard G. Stanley and Andrew G. Elefanty
DOI: 10.1242/dev.193037

Editor: Gordon Keller

Review timeline

Original submission:	19 May 2020
Editorial decision:	3 July 2020
First revision received:	26 August 2020
Accepted:	24 September 2020

Original submission

First decision letter

MS ID#: DEVELOP/2020/193037

MS TITLE: Human yolk sac-like haematopoiesis generates RUNX1- and GFI1/1B-dependent blood and SOX17-positive endothelium

AUTHORS: Freya F Bruveris, Elizabeth S Ng, Ana Rita Leuitoguinho, Ali Motazedian, Katerina Vlahos, Koula Sourris, Robyn Mayberry, Penelope McDonald, Lisa Azzola, Nadia M Davidson, Alicia Oshlack, Edouard G Stanley, and Andrew G Elefanty

I have now received all the referees reports on the above manuscript, and have reached a decision. The referees' comments are appended below, or you can access them online: please go to BenchPress and click on the 'Manuscripts with Decisions' queue in the Author Area.

The overall evaluation is positive and we would like to publish a revised manuscript in Development, provided that the referees' comments can be satisfactorily addressed. Please attend to all of the reviewers' comments in your revised manuscript and detail them in your point-by-point response. If you do not agree with any of their criticisms or suggestions explain clearly why this is so.

We are aware that you may currently be unable to access the lab to undertake experimental revisions. If it would be helpful, we encourage you to contact us to discuss your revision in greater detail. Please send us a point-by-point response indicating where you are able to address concerns raised (either experimentally or by changes to the text) and where you will not be able to do so within the normal timeframe of a revision. We will then provide further guidance. Please also note that we are happy to extend revision timeframes as necessary.

Reviewer 1

Advance summary and potential significance to field

This paper builds on pioneering studies originally in murine ES cells and subsequently in mouse embryos and human ES cells describing the initial emergence of hematopoiesis from a

© 2020. Published by The Company of Biologists under the terms of the Creative Commons Attribution License (https://creativecommons.org/licenses/by/4.0/).

'hemangioblast' precursor that is capable of generating both endothelial cells and blood cells. As such, the authors focus on "extraembryonic", that is, yolk sac-derived, hematopoiesis rather than "intraembryonic", aorta-derived hematopoiesis, which leads to HSC generation.

The authors make use of previously developed dual reporter (Sox17, Runx1c) human iPS cells to identify presumptive endothelial cells and blood cells, respectively. Using this system in conjunction with colony assays in methylcellulose (Blast-CFC assay), the authors identify a Sox17-negative hemangioblast and tease apart the subsequent emergence of hematopoietic and endothelial fates. Transcriptional data support the concept that these fates represent binary decisions downstream of a hemangioblast, though clonal studies were not performed. Additional experiments examine the function of Runx1c and of Gfi1b in blood cell emergence using knockout and chemical inhibition approaches, respectively, which confirm previous studies, primarily in the murine system, that Gfi1b is necessary for all blood cell emergence, while Runx1 is necessary for the continued generation of blood cells except for an initial population of (primitive) erythroid cells. Overall, the work is very carefully performed, statistically analyzed, and logically laid out. Extensive supplementary data are provided to support the findings presented in the body of the text. This paper advances our understanding of the initial phases of blood cell emergence in the human system.

Comments for the author

Some minor issues should be addressed:

1.Lines 135-139: reculturing experiments reveal an erythroid bias to the initial emergence of blood cells. It is not clear how the authors know that the CD34+CD43+ cells yielded more "myeloid cells", since no myeloid markers are evident in Figures 1I and S1B.

2.Lines 237-240: Given the persistence of primitive erythropoiesis but the lack of myeloid cells and platelets in Runx1-null mouse embryos, was there a larger decrease in the expression of genes associated with myelopoiesis and megakaryopoiesis (vs. the genes associated with erythropoiesis) in the Runx1-KO cells?

3.Line 244: might better read: "..“the initial wave of" yolk sac erythroid differentiation', since definitive erythroid progenitors also emerge in the yolk sac of murine and human embryos.

4. It is interesting that the authors needed to drastically modify the differentiation conditions (CHIR, cytokines, culture at the air:liquid interface) to analyze the role of Runx1c in primitive erythropoiesis. The appearance of highly hemoglobinized cells by day 20 of culture of the Runx1c-KO cells is striking (figure 4B). Why are the Runx1c-null erythroid cells so much more hemoglobinized compared to the cultures of normal iPS cells? Is it that they persist longer (as evidenced by the increased GYPA expression at days 18 and beyond). Runx1 has been shown to regulate the terminal maturation of primitive erythroid cells in the mouse, with Runx1-null primitive erythroblasts exprressing lass Gata1, less Ter119 (glycophorin A), and have altered membrane morphology (Yokomizo, Blood 2008). Were any defects in the Runx1c-KO erythroid cells noted?

5.Lines 261-262: How do you know that the Runx1c-KO cells differentiate "only" into erythroid cells, since no examination of other lineages was reported? How do you know that the CD43+ cells in Fig. 4E are "myeloid", as described in the Figure legend, since no lineage-specific markers of myeloid cells was employed? Might the GYPA-negative cells also represent megakaryocytes? Given the experimental data it might be better to use the term 'non-erythroid' rather than myeloid.

<u>Reviewer 2</u>

Advance summary and potential significance to field

In this manuscript entitled "Human yolk sac-like hematopoiesis generates RUNX1- and GFI1/1B-dependent blood and SOX17-positive endothelium," the authors explore the genetic regulatory network controlling early human blood development. Their main conclusion is that human blood

© 2020. Published by The Company of Biologists under the terms of the Creative Commons Attribution License (https://creativecommons.org/licenses/by/4.0/).

cell development arises from a hemangioblast-like SOX17- endothelial cell, and is sequentially dependent upon GFI1/1B and RUNX1.

The manuscript is well organized, and the results very interesting and important. The experiments presented are well performed, conclusive, and the interpretations sound. Although the findings presented are not entirely unexpected, given the results previously obtained in the mouse, they are definitely worth publication as they were never shown in the human setting.

Comments for the author

I have only a few comments and suggestions to expand/improve the manuscript.

1. The authors interpret the observation that the complete loss of hematopoiesis in the presence of LSD1 inhibition in the human model as a suggestion that the human extraembryonic yolk sac-like culture is more similar to the mouse AGM rather than yolk sac hemogenic endothelium (lines 390-393). This could be an overinterpretation, as the mouse findings were obtained in the double knockout Gfi1/1b embryos, whereas the human results were generated with the LSD1 inhibitor. As such, the differences in results might reflect a broader phenotype with the inhibitor than with the double Gfi1/1b knockout, and not a fundamental difference between human and mouse yolk sac blood development.

2. Following on this, the phenotype observed upon LSD1 inhibitor treatment could be further explored. It would be interesting to perform LSD1 inhibitor treatment on the dual reporter line to determine the phenotype of the endothelial cells obtained upon LSD1 inhibition. Are they acquiring Sox17 expression or remaining Sox17-? Is the inhibition reversible?

3. The authors suggest that GFI1/1B may be a regulator, as well as a target, of RUNX1, based on the lower levels of RUNX1 transcripts in cultures treated with LSD inhibitor (lines 298-301). Another interpretation is that upon inhibition of LSD1, the expression of SOX7 (and perhaps SOX17) might not be downregulated.
SOX7 has been shown to hinder RUNX1 DNA binding (PMID 27802172 and 29530939) and alter its transcriptional activity, and by so downregulates its expression, as RUNX1 activity regulates its own expression.

<u>Reviewer 3</u>

Advance summary and potential significance to field

In vitro differentiation of human pluripotent stem cells (hPSCs) provides a valuable model system to study human developmental hematopoiesis as the study of primary material has practical and ethical restrictions. Here, the manuscript by Bruveris et al reports on the emergence of human vascular and hematopoietic lineages from hPSCs, using a combination of functional and molecular analyses. The authors are experts in the field and show that the earliest human yolk sac-like blood cells derive from SOX17- endothelial cells, and that most human blood production, with the exception of primitive erythrocytes, was dependent on RUNX1. Furthermore, blocking GFI1/1B activity abrogated all blood formation. The study provides important data that show conservation of the early requirements for these transcription factors between human and mouse.

Comments for the author

I have strong reservations about the conclusion that SOX17- endothelium is hemangioblast-like and is the major source of SOX17+ endothelium. This should be supported by stronger data. If those are not available, the model in Figure 6 should in my view be adapted to reflect this.

Main concerns

1. To my understanding, the authors base the conclusion that d2 SOX17- endothelium gives rise to SOX17+ endothelium on two pieces of information: the presences of a minor population of SOX17+ endothelium in re-cultures of SOX17- cells (Fig 1I, K) and on the RNA-seq data (Fig 2 and corresponding Suppl figure) which is interpreted as arguing “for the presence of a binary ‘switch’

© 2020. Published by The Company of Biologists under the terms of the Creative Commons Attribution License (https://creativecommons.org/licenses/by/4.0/).

active in the d2 SOX17-ENDO cells that will lead to either a haematopoietic or endothelial fate, and are consistent with a haemangioblast-like function of these cells.” (ln194-196). The low number of SOX17+ cells in the re-cultures could alternatively be explained as originating from contaminating SOX17+ cells. Purities are not stated in the paper, but even a 99% purity could be problematic as SOX17+ cells seem to efficiently give rise to more SOX17+ cells (Fig 1). Regarding the ‘binary switch’, how does the RNA-seq data suggest this? Could this be explained differently/better? Should this not be demonstrated by clonal assays? The re-culture data does not seem to support SOX17-endothelium being a major source of SOX17+ endothelium (as stated in e.g. line 403).

2. In a similar vein, the few CD43+ cells generated in SOX17+ endothelium cultures could be the result of contaminating SOX17- cells. The gating strategy suggest this is not implausible (FigS1C). This possibility is further supported by the near identical expression signatures and clonogenic potential for the CD34+CD43+ cells derived from SOX17- and SOX17+ cultures (FIgS1D-I, line 164-166), and the lack of a hemogenic/hematopoietic signature in d3 S17+ ENDO (Fig 2F).

3. Given the concerns in point 1 and 2, could it be that the SOX17- endothelial cell population contains progenitors that are (i) equivalent to the yolk sac blood island mesoderm that gives rise to primitive erythrocytes, and (ii) contains other cells that are similar to yolk sac hemogenic endothelium giving rise to EMPs. In this interpretation, the SOX17+ endothelium would represent a separate population of arterial endothelium (possibly including prospective intraembryonic hemogenic endothelium?). The SOX17- cells corresponding with the yolk sac blood island mesoderm (which in mouse is known to express several endothelial markers) also reconciles this study with a study from the Keller group which showed that in mouse the hemangioblast is located in the primitive streak.

Minor concerns

- The manuscript is data-rich and assesses different time points and different cell populations, as is required when studying a developmental process. However, at times it is challenging for the reader to see the different pieces of data that support a text section as several figures need to be compared. Could the layout of the figures be revisited to help the reader (e.g. the order of the panels on the page is not always intuitive)? Could the rationale for the different culture systems be made clearer? The color coding for SOX17 and RUNX1 is helpful, but is not used throughout.

- Ln54: there are studies of early blood development in human embryos. If this sentence mainly refers to genetically tractable systems, this could be clarified.

- Fig 1B: the red dots are hard to see – this could be mitigated by increasing dot size (similar for some other plots).

- Fig 1C: the data are from 5 experiments so the average and variation should be reported – if these are representative dotplots for Fig 1F (showing the individual values), it would be helpful to mention that.

- The gates for CD34- cells in Fig 1H and S1A are not the same. What was used?

- Fig 1J and K: a nuclear counterstain could be helpful.

- Ln 229: would be good to specify this is ‘in human’.

- Ln 236: The percentage in the KO seems not changed much, but the wild type does increase. So it may be clearer to say "while in wild type an increase in CD43+ cells was seen, no such cells developed in KO cultures."

- Figure 4F: It is not clear these are erythroid colonies as stated in the text.

- I may have missed this, but did not see a list of Taqman probe sets.

© 2020. Published by The Company of Biologists under the terms of the Creative Commons Attribution License (https://creativecommons.org/licenses/by/4.0/).

First revision

Author response to reviewers' comments

Reviewer 1 Advance Summary and Potential Significance to Field:
This paper builds on pioneering studies originally in murine ES cells and subsequently in mouse embryos and human ES cells describing the initial emergence of hematopoiesis from a 'hemangioblast' precursor that is capable of generating both endothelial cells and blood cells. As such, the authors focus on "extraembryonic", that is, yolk sac-derived, hematopoiesis rather than "intraembryonic", aorta-derived hematopoiesis, which leads to HSC generation.

The authors make use of previously developed dual reporter (Sox17, Runx1c) human iPS cells to identify presumptive endothelial cells and blood cells, respectively. Using this system in conjunction with colony assays in methylcellulose (Blast-CFC assay), the authors identify a Sox17-negative hemangioblast and tease apart the subsequent emergence of hematopoietic and endothelial fates. Transcriptional data support the concept that these fates represent binary decisions downstream of a hemangioblast, though clonal studies were not performed. Additional experiments examine the function of Runx1c and of Gfi1b in blood cell emergence using knockout and chemical inhibition approaches, respectively, which confirm previous studies, primarily in the murine system, that Gfi1b is necessary for all blood cell emergence, while Runx1 is necessary for the continued generation of blood cells except for an initial population of (primitive) erythroid cells. Overall, the work is very carefully performed, statistically analyzed, and logically laid out. Extensive supplementary data are provided to support the findings presented in the body of the text. This paper advances our understanding of the initial phases of blood cell emergence in the human system.

Reviewer 1 Comments for the Author:
Some minor issues should be addressed:
1. Lines 135-139: reculturing experiments reveal an erythroid bias to the initial emergence of blood cells. It is not clear how the authors know that the CD34+CD43+ cells yielded more "myeloid cells", since no myeloid markers are evident in Figures 1I and S1B.

The statement made in the text was: "Re-culturing the haematopoietic populations yielded predominantly GYPA+ erythroid cells (76.1±4.1%, n=3) from the more mature CD34-CD43+ fraction whilst the CD34+CD43+ fraction yielded more RUNX1C+ myeloid cells (65.7±6.8%, n=3) (Fig. 1I and Fig. S1B), suggesting an erythroid bias in the earliest blood cells produced from blast colonies."
Response: We assigned erythroid vs myeloid lineage based on the immunophenotype. Cells that were $GYPA^{+}CD43^{+}RUNX1C^{-}$ were designated to be maturing erythroid cells, since GYPA is uniquely expressed in this lineage and RUNX1 is downregulated as erythroid cells mature (Kuvardina et al., Blood 125:3570-3579, 2015; Willcockson et al., PNAS 116: 17841-17847, 2019). Myeloid lineage was assigned on the basis of the $RUNX1C^{+}43^{+}GYPA^{-}$ phenotype. RUNX1 is expressed in myeloid cells and megakaryocytes as they mature and actively represses erythroid differentiation (Kuvardina 2015; Willcockson 2019). Pre-empting a later comment by this referee, we acknowledge that they are correct in stating that RUNX1 is expressed in megakaryocytes (Kuvardina et al., Blood 125:3570-3579, 2015) and therefore that the $RUNX1C^{+}$ cells should more accurately be described as 'non-erythroid'. This has been modified in the text.

Re-worded text (lines 135-139): "Re-culturing the haematopoietic populations yielded predominantly $GYPA^{+}$ erythroid cells (76.1±4.1%, n=3) from the more mature $CD34^{-}CD43^{+}$ fraction whilst the $CD34^{+}CD43^{+}$ fraction yielded more $RUNX1C^{+}$ non-erythroid (myeloid and megakaryocytic) cells (65.7±6.8%, n=3) (Fig. 1I and Fig. S1B), suggesting an erythroid bias in the earliest blood cells produced from blast colonies."

2. Lines 237-240: Given the persistence of primitive erythropoiesis but the lack of myeloid cells and platelets in Runx1-null mouse embryos, was there a larger decrease in the expression of genes associated with myelopoiesis and megakaryopoiesis (vs. the genes associated with erythropoiesis) in the Runx1-KO cells?

The statement made in the text was: "Gene expression confirmed the down regulation of RUNX1,

© 2020. Published by The Company of Biologists under the terms of the Creative Commons Attribution License (https://creativecommons.org/licenses/by/4.0/).

RUNX1C and GFI1B, with similarly reduced expression of erythroid lineage genes (GATA1, KLF1, ε-GLOBIN and γ-GLOBIN) and increased expression of endothelial SOX17 and CD34 (Fig. 3H and Fig. S3F)."

Response: Supporting evidence for the erythroid restriction of *RUNX1*-KO haematopoiesis was provided by RNA seq analysis at d6 of differentiation. At this early time point there were only 17 differentially expressed genes between the parental SOX-RUNX and the *RUNX1*-KO cultures (Supplementary Tables 5 and 7). Notably, 5 of the 10 genes downregulated in the d6 *RUNX1*-KO cell line are expressed in megakaryocytes, myeloid cells or B cells (*HDC*, *GCSAML*, *PLEK*, *RGS18*, *TSPAN33*) consistent with the hypothesis that differentiation to non-erythroid lineages is compromised. Conversely, 3 of 7 genes with increased expression in *RUNX1*-KO cells are expressed in erythroid cells (*SLC4A1*, *HBA2*, *HEMGN*) arguing for a complementary increase in erythroid differentiation, as indeed was observed under these modified differentiation conditions.

This additional data has been incorporated into the manuscript in lines 288-296, and new **Supplementary Table 7**.

3. Line 244: might better read: "..“the initial wave of” yolk sac erythroid differentiation', since definitive erythroid progenitors also emerge in the yolk sac of murine and human embryos.

Re-worded text (Lines 260-262): "The presence of abundant nucleated erythroid cells in *Runx1*-knock out mouse embryos at E12.5 (Okuda et al., 1996; Wang et al., 1996) argues that the initial wave of yolk sac erythroid differentiation remains intact…"

4. It is interesting that the authors needed to drastically modify the differentiation conditions (CHIR, cytokines, culture at the air:liquid interface) to analyze the role of Runx1c in primitive erythropoiesis. The appearance of highly hemoglobinized cells by day 20 of culture of the Runx1c-KO cells is striking (figure 4B). Why are the Runx1c-null erythroid cells so much more hemoglobinized compared to the cultures of normal iPS cells? Is it that they persist longer (as evidenced by the increased GYPA expression at days 18 and beyond). Runx1 has been shown to regulate the terminal maturation of primitive erythroid cells in the mouse, with Runx1-null primitive erythroblasts exprressing lass Gata1, less Ter119 (glycophorin A), and have altered membrane morphology (Yokomizo, Blood 2008). Were any defects in the Runx1c-KO erythroid cells noted?

Response: To clarify, our *RUNX1*-KO cells do not express any of the *RUNX1* isoforms, they are not just *RUNX1C* null. Indeed, we have previously generated and published *RUNX1C*-null hPSC lines as part of another study and did not observe an obvious phenotype (Ng et al., Nature Biotechnology, 2016). We agree that the results of the *RUNX1*-KO were striking and we believe these results are in keeping with the recent literature dissecting the role of *RUNX1* and interacting transcription factors on erythroid, myeloid and megakaryocytic development.

The following comments have been inserted into the manuscript, lines 315-322: "*RUNX1* and *SPI1* are expressed in erythroid progenitor cells, but their levels decline with erythroid maturation. The decline in *RUNX1* is the major driver of the reduction in *SPI1* expression (Willcockson et al., 2019). Both *RUNX1* and *SPI1* repress key erythroid transcription factors, exemplified by *KLF1* (Kuvardina et al., 2015), and the enforced expression of either gene prevents terminal erythroid differentiation (Willcockson et al., 2019). We hypothesise that in the absence of *RUNX1* to drive myeloid and megakaryocytic differentiation, *SPI1* levels also eventually fall, and the wave of $GYPA^{+}CD43^{+}$ cells seen at d7 (Fig. 5C) defaults entirely to erythroid lineage differentiation. This may explain the greater quantity of primitive erythroid cells generated from *RUNX1*-KO cells."

We have not compared the lifespans of erythroid cells derived from wild type and *RUNX1*-deleted cells, nor have we investigated possible defects in terminal maturation of *RUNX1*-KO erythroid cells.

5. Lines 261-262: How do you know that the Runx1c-KO cells differentiate "only" into erythroid cells, since no examination of other lineages was reported? How do you know that the CD43+ cells in Fig. 4E are "myeloid", as described in the Figure legend, since no lineage-specific markers of myeloid cells was employed? Might the GYPA-negative cells also represent megakaryocytes? Given the experimental data it might be better to use the term 'non-erythroid' rather than myeloid.

© 2020. Published by The Company of Biologists under the terms of the Creative Commons Attribution License (https://creativecommons.org/licenses/by/4.0/).

The statement made in the text was: "These data indicate that *RUNX1* is not required for the generation of the first CD43-expressing cells that subsequently differentiate only to erythroid cells."

Response: As far as we are aware, GYPA expression is restricted to erythroid cells, and a single wave of $GYPA^{+}$ cells are the only blood cells seen in the *RUNX1*-KO differentiations. Furthermore, as indicated in our RNA seq analysis, by d6 of differentiation expression of megakaryocyte genes is diminished in the *RUNX1*-KO samples. However, as indicated in our responses to the preceding questions from this reviewer, we acknowledge that the GYPA-negative cells that express *RUNX1C* in the parental cell line would probably include megakaryocyte precursors.

Reviewer 2 Advance Summary and Potential Significance to Field:
In this manuscript entitled "Human yolk sac-like hematopoiesis generates RUNX1- and GFI1/1B-dependent blood and SOX17-positive endothelium," the authors explore the genetic regulatory network controlling early human blood development. Their main conclusion is that human blood cell development arises from a hemangioblast-like SOX17- endothelial cell, and is sequentially dependent upon GFI1/1B and RUNX1.

The manuscript is well organized, and the results very interesting and important. The experiments presented are well performed, conclusive, and the interpretations sound. Although the findings presented are not entirely unexpected, given the results previously obtained in the mouse, they are definitely worth publication as they were never shown in the human setting.

Reviewer 2 Comments for the Author:
I have only a few comments and suggestions to expand/improve the manuscript.
1. The authors interpret the observation that the complete loss of hematopoiesis in the presence of LSD1 inhibition in the human model as a suggestion that the human extraembryonic yolk sac-like culture is more similar to the mouse AGM, rather than yolk sac hemogenic endothelium (lines 390- 393). This could be an overinterpretation, as the mouse findings were obtained in the double knockout Gfi1/1b embryos, whereas the human results were generated with the LSD1 inhibitor. As such, the differences in results might reflect a broader phenotype with the inhibitor than with the double Gfi1/1b knockout, and not a fundamental difference between human and mouse yolk sac blood development.

The statement made in the text was: "In our human pluripotent stem cell model, the complete loss of haematopoiesis in the presence of LSD1 inhibition suggests that the human extra embryonic, yolk sac-like culture modelled in our hPSC in vitro differentiation is more similar to the mouse AGM, rather than yolk sac haemogenic endothelium."

Response: The reviewer makes a good point, and we acknowledge that the differences may possibly reflect a more severe phenotype with the LSD1 inhibitor than was seen in the mouse double Gfi1/1b knockout. We have therefore re-worded the text to reflect this.

Re-worded text (lines 434-439): "In our human pluripotent stem cell model, the complete loss of haematopoiesis in the presence of LSD1inhibition suggests that the human extra embryonic, yolk sac- like culture modelled in our hPSC in vitro differentiation may be more similar to the mouse AGM, rather than yolk sac haemogenic endothelium. However, an important caveat remains that the effects of the chemical inhibitor may differ slightly from the results that would be obtained from a double knock-out of GFI1/1B genes."

2. Following on this, the phenotype observed upon LSD1 inhibitor treatment could be further explored. It would be interesting to perform LSD1 inhibitor treatment on the dual reporter line to determine the phenotype of the endothelial cells obtained upon LSD1 inhibition. Are they acquiring Sox17 expression or remaining Sox17-? Is the inhibition reversible?

Response: We have examined the phenotype of the endothelium generated under LSDi conditions in the BL-CFC assay and compared these with endothelium produced by the *RUNX1*-KO cells and the SOX-RUNX parental cell line. After 10 days differentiation in methylcellulose, there were very few endothelial cells ($CD34^{+}CD31^{+}KDR^{+}CD43^{-}$) in the SOX-RUNX cultures (<1%) and these were >95% $SOX17^{+}$. There were very few viable cells in the *RUNX1*-KO cultures, but approximately 40%

© 2020. Published by The Company of Biologists under the terms of the Creative Commons Attribution License (https://creativecommons.org/licenses/by/4.0/).

were endothelial cells, almost uniformly SOX17$^+$. The LSDi treated SOX-RUNX cells presented a very similar phenotype to the *RUNX1*-KO cells, except that a few CD43$^+$ cells remained in the *RUNX1*-KO. The LSDi treated SOX-RUNX endothelial cells were also SOX17$^+$.

This data is included in the text, lines 334-339 and shown in new Figure S6A, B: "The endothelium generated under LSDi conditions in the blast colony assay was very similar to endothelium produced by the *RUNX1*-KO cells and the SOX-RUNX parental cell line. After 10 days differentiation in methylcellulose, there were very few endothelial cells (CD34$^+$CD31$^+$KDR$^+$CD43$^-$) in the SOX RUNX cultures (<1%) and these were >95% SOX17$^+$. In the *RUNX1*-KO and the LSDi treated SOX-RUNX cultures, approximately 30-40% of the viable cells were SOX17$^+$ endothelial cells (Fig. S6A, B)."
We have not explored the reversibility of the LSD1 inhibition.

3. The authors suggest that GFI1/1B may be a regulator, as well as a target, of RUNX1, based on the lower levels of RUNX1 transcripts in cultures treated with LSD inhibitor (lines 298-301). Another interpretation is that upon inhibition of LSD1, the expression of SOX7 (and perhaps SOX17) might not be downregulated. SOX7 has been shown to hinder RUNX1 DNA binding (PMID 27802172 and 29530939) and alter its transcriptional activity, and by so downregulates its expression, as RUNX1 activity regulates its own expression.

The statement made in the text was: "PCR analysis indicated that levels of *RUNX1* transcripts were lower in cultures treated with LSDi (compare SOX-RUNX with and without LSDi in Fig. 5K and Fig. S3G), suggesting that GFI1/1B may be a regulator, as well as a target, of RUNX1."

Response: Examination of our d4 and d6 RNA seq analysis comparing SOX-RUNX with and without LSD1 inhibition does not reveal any statistically significant difference in the levels of *SOX7* or *SOX17* expression between the samples.
The reviewer argues that a failure of *SOX7* and *SOX17* to be downregulated may be responsible for the diminution in *RUNX1* transcription. We are happy to entertain alternative explanations for the phenomenon that we observed, however the literature cited by the reviewer (Lilly et al., Development 2016; Lie-A-Ling et al., Development 2018) explicitly state that SOX7 protein interacts with RUNX1 protein, negatively influencing expression of its target genes, without affecting RUNX1 transcription. This does not seem to support the alternative hypothesis raised by the reviewer.

Reviewer 3 Advance Summary and Potential Significance to Field:
In vitro differentiation of human pluripotent stem cells (hPSCs) provides a valuable model system to study human developmental hematopoiesis as the study of primary material has practical and ethical restrictions. Here, the manuscript by Bruveris et al reports on the emergence of human vascular and hematopoietic lineages from hPSCs, using a combination of functional and molecular analyses. The authors are experts in the field and show that the earliest human yolk sac-like blood cells derive from SOX17- endothelial cells, and that most human blood production, with the exception of primitive erythrocytes, was dependent on RUNX1. Furthermore, blocking GFI1/1B activity abrogated all blood formation. The study provides important data that show conservation of the early requirements for these transcription factors between human and mouse.

Reviewer 3 Comments for the Author:
I have strong reservations about the conclusion that SOX17- endothelium is hemangioblast-like and is the major source of SOX17+ endothelium. This should be supported by stronger data. If those are not available, the model in Figure 6 should in my view be adapted to reflect this.

Response: We have provided additional data, detailed below, that we believe strongly supports our assertion that SOX17$^-$ endothelium is the major source of SOX17$^+$ endothelium.

Main concerns
1. To my understanding, the authors base the conclusion that d2 SOX17- endothelium gives rise to SOX17+ endothelium on two pieces of information: the presences of a minor population of SOX17+ endothelium in re-cultures of SOX17- cells (Fig 1I, K) and on the RNA-seq data (Fig 2 and corresponding Suppl figure) which is interpreted as arguing "for the presence of a binary 'switch' active in the d2 SOX17-ENDO cells that will lead to either a haematopoietic or endothelial fate,

© 2020. Published by The Company of Biologists under the terms of the Creative Commons Attribution License (https://creativecommons.org/licenses/by/4.0/).

and are consistent with a haemangioblast-like function of these cells." (ln194-196). The low number of SOX17+ cells in the re-cultures could alternatively be explained as originating from contaminating SOX17+ cells. Purities are not stated in the paper, but even a 99% purity could be problematic as SOX17+ cells seem to efficiently give rise to more SOX17+ cells (Fig 1). Regarding the 'binary switch', how does the RNA-seq data suggest this? Could this be explained differently/better? Should this not be demonstrated by clonal assays? The re-culture data does not seem to support SOX17- endothelium being a major source of SOX17+ endothelium (as stated in e.g. line 403).

Response: The initial piece of data indicating that SOX17+ endothelium derived from SOX17- endothelium was the observed kinetics of surface marker and reporter acquisition during differentiation shown in Figure 1F.
Specifically, we state in the text (**lines 120-122**): " SOX17 (11.4±3.1%, n=5) and CD43 (16.5±3.6%, n=5) expression appeared after two days of methylcellulose differentiation, delineating subsets of endothelial ($SOX17^+CD34^+CD43^-$) and haematopoietic ($CD43^+$) cells". These findings indicate that the $SOX17^+$ cells arose from a $SOX17^-$ culture, in a largely mutually exclusive fashion with the appearance of $CD43^+$ blood cells. The presence of the minor population of $SOX17^+$ cells in the re-culture experiments was not argued to support the derivation of $SOX17^+$ cells from the $SOX17^-$ ancestor. Rather, at d3 of analysis, we stated that "…most of the SOX17- cells remained mCHERRY negative, suggesting that the allocation of cells to a $SOX17^+$ fate was largely complete by d3 in methylcellulose. " (**lines 146-147**).

The data pertaining to the 'binary switch' interpretation of the RNA seq data is discussed in the paragraph beginning with the sentence (line 201): " Patterns of differentially expressed genes between haematopoietic and endothelial cells were also consistent with cells segregating into distinct *SOX17*- expressing endothelium or $CD34^+CD43^+$ haematopoietic fates during blast colony differentiation (Fig. S2C)." Descriptions of these gene expression patterns are then "…summarised to state that the same genes up regulated during the transition from d2 $SOX17^-$ENDO to d3 $SOX17^+$ENDO are down regulated in the transition to $CD34^+CD43^+$, and vice-versa." It was based on these reciprocal patterns of gene expression between the transitions to $SOX17^+$ endothelium or to $CD34^+CD43^+$ blood cells, that we believed argued "…for the presence of a binary 'switch' active in the d2 $SOX17^-$ENDO cells that will lead to either a haematopoietic or endothelial fate, and are consistent with a haemangioblast- like function of these cells…"(**line 211-213**).

However, we are careful to point out that "… Our study indicated that the d2 $SOX17^-$ENDO was the precursor of both $CD34^+CD43^+$ haematopoietic cells and a distinct $SOX17^+$ expressing endothelium, although we have not shown that one cell could give rise to both progeny…" (**line 398-401**). We have also included this qualification in the legend to Figure 7.

We believe that the single cell deposition data (Fig. 1N) argues most persuasively that the hematopoietic cells arise from a $SOX17^-$ endothelial precursor, both at day 2 and at day 3 of methylcellulose culture.

Therefore, to support our argument that the $SOX17^+$ endothelium also arises from a SOX17- endothelial precursor, and to address the reviewer's concerns, we performed live cell imaging of $SOX17^-CD34^+CD43^-$ endothelium sorted from d2 methylcellulose cultures (d2 $SOX17^-$ENDO) and re-plated into an endothelial network assay. A movie comprising a time lapse series of images taken at 10 minute intervals for 65 hours is presented (**Supplementary Movie 1**) and single images and analyses are provided in new **Figure 2**, with images from a second independent experiment provided in **Supplementary Figure 2**. It can be seen from the images (Supplementary Movie 1 and Figure 2C) that the endothelia were initially $SOX17^-$, and that individual, $SOX17^-$ cells began to acquire expression of the SOX17-mCHERRY reporter after 6 hours of observation. Importantly, the cells acquired SOX17-mCHERRY expression by 24-28 hours, and during this period there was little increase in cell numbers, precluding division of contaminating $SOX17^+$ cells as the reason for the increase in $SOX17^+$ cell numbers (Figure 2D). Indeed, after this period the number of blood cells rapidly increased and the number of $SOX17^+$ cells decreased a little and stabilised. Similar results were observed in a second experiment (Supplementary Figure 2). These data strongly support the premise that d3 $SOX17^+$ENDO derives from the same d2 $SOX17^-$ENDO precursor population that also exhibits high haemogenic activity (Fig. 1N).

© 2020. Published by The Company of Biologists under the terms of the Creative Commons Attribution License (https://creativecommons.org/licenses/by/4.0/).

These arguments are included in the text, **lines 159-172**.

2. In a similar vein, the few CD43+ cells generated in SOX17+ endothelium cultures could be the result of contaminating SOX17- cells. The gating strategy suggest this is not implausible (FigS1C). This possibility is further supported by the near identical expression signatures and clonogenic potential for the CD34+CD43+ cells derived from SOX17- and SOX17+ cultures (FIgS1D-I, line 164-166), and the lack of a hemogenic/hematopoietic signature in d3 S17+ ENDO (Fig 2F).

Response: We are confident, for reasons enunciated in the manuscript, that this finding was not the result of imperfect gating at the time of sorting. Indeed, we tried hard to convince ourselves that the infrequent clonogenic cells found in the SOX17$^+$ population were the result of such an artifact. However, we showed convincingly (Figure S3) that the levels of *SOX17* and *mCHERRY* RNA expression were up to 30-fold higher in the SOX17-mCHERRY sorted endothelium and blood cells than in their CHERRY reporter negative counterparts. If the results had been the consequence of a sorting error, the expression of *SOX17* and of *mCHERRY* RNAs would not have differed between the samples. Furthermore, consistent with the greater haemogenic capacity of the SOX17-negative endothelium, the key haematopoietic transcription factors *RUNX1*, *GFI1* and *SPI1* were expressed more highly in these SOX17-negative endothelial cells. However, in the blood cells emerging from either endothelium, expression of these transcription factors did not differ, nor did globin chain expression, clonogenic capacity or distribution of myeloid or erythroid colonies.

We have made this point more clearly in the text, **lines 175-179**.

3. Given the concerns in point 1 and 2, could it be that the SOX17- endothelial cell population contains progenitors that are (i) equivalent to the yolk sac blood island mesoderm that gives rise to primitive erythrocytes, and (ii) contains other cells that are similar to yolk sac hemogenic endothelium giving rise to EMPs.

Response: We believe that we have adequately dealt with the concerns raised by the reviewer in points 1 and 2. However, we do agree with the reviewer that the SOX17$^-$ endothelium harbours progenitors that can generate primitive erythrocytes and those that are more similar to yolk sac endothelium that gives rise to EMPs. We do not know if the proportions of SOX17$^-$ endothelial precursors allocated to primitive or EMP fates are fixed, or whether there is plasticity in the distribution. The observation that in the absence of RUNX1, there is greater primitive erythroid differentiation than in the control cell line does suggest that there may be some plasticity in the allocation of GYPA$^+$CD43$^+$ precursors to different fates. However, this is speculative and would need to be addressed in a future study.

In this interpretation, the SOX17+ endothelium would represent a separate population of arterial endothelium
Response: Yes, we would agree that the endothelium expressing SOX17 is likely to be arterial but of extra embryonic, yolk sac type.

(possibly including prospective intraembryonic hemogenic endothelium?).
Response: We do not believe so. The gene expression profile of SOX17$^+$ENDO shows that it is *HOXA*$^-$ (Figure S4F), so this is yolk sac type endothelium.

The SOX17- cells corresponding with the yolk sac blood island mesoderm (which in mouse is known to express several endothelial markers) also reconciles this study with a study from the Keller group which showed that in mouse the hemangioblast is located in the primitive streak.
Response: We agree. We also showed that the BL-CFC is in a streak-like population - this is the phenotype of the input cells for the methylcellulose BL-CFC assay. The transcriptional profiling of the population that seeded the methylcellulose demonstrated expression of primitive streak like markers, and also KDR and APLNR (Figure 3E, F) (**lines 215-218**).

Minor concerns
-The manuscript is data-rich and assesses different time points and different cell populations, as is required when studying a developmental process. However, at times it is challenging for the reader to see the different pieces of data that support a text section as several figures need to be

© 2020. Published by The Company of Biologists under the terms of the Creative Commons Attribution License (https://creativecommons.org/licenses/by/4.0/).

compared. Could the layout of the figures be revisited to help the reader (e.g. the order of the panels on the page is not always intuitive)? Could the rationale for the different culture systems be made clearer? The color coding for SOX17 and RUNX1 is helpful, but is not used throughout.

Response: We appreciate the difficulty in navigating a complex series of experiments and data. We have reviewed the organisation, labelling and colour coding of the figures and tried to rationalise these to make it easier and more intuitive for the reader.

-Ln54: there are studies of early blood development in human embryos. If this sentence mainly refers to genetically tractable systems, this could be clarified.
Response: We have clarified the statement to read (**lines 54-56**): "…Since studies of genetically modified human blood cells in the context of a developing human embryo are not possible, haematopoietic differentiation of human pluripotent stem cells has emerged as the most tractable surrogate experimental system…"

-Fig 1B: the red dots are hard to see – this could be mitigated by increasing dot size (similar for some other plots).
Response: We appreciate the difficulty in visualising dot plots where there are small numbers of cells. Unfortunately, the flow cytometry analysis software that we have available does not offer the option of increasing dot size. In selected cases we have tried displaying the data as contour plots to overcome this problem. Although these plots are also less than perfect, they do allow the reader to see that there are events displayed. However, we have chosen not to change all the FACS plots to contours, because these plots do compromise the ability to visualise the colour coding of the reporter cells.

-Fig 1C: the data are from 5 experiments so the average and variation should be reported – if these are representative dot plots for Fig 1F (showing the individual values), it would be helpful to mention that.
Response: In this figure and all other similar panels, the mean, SEM and number of experiments is reported in the text. We chose not to repeat these data on the figures to reduce clutter. We have indicated in the figure legend that the mean and SEM are reported in the text.

-The gates for CD34- cells in Fig 1H and S1A are not the same. What was used?
Response: The gating strategy shown in Fig. 1H was the one used. Figure S1A has been corrected.

-Fig 1J and K: a nuclear counterstain could be helpful.
Response: Unfortunately, we could not provide a nuclear counterstain for a live cell assay. However, we have included an overlaid image as an alternative to make it easier to appreciate the reporter positive and negative cells.

-Ln 229: would be good to specify this is 'in human'.
Response: We have modified the text to read (**line 246**): "… To examine whether RUNX1 is a key driver of the EHT in human extra embryonic, yolk sac-like haematopoiesis…"

-Ln 236: The percentage in the KO seems not changed much, but the wild type does increase. So it may be clearer to say "while in wild type an increase in CD43+ cells was seen, no such cells developed in KO cultures."
Response: We have reworded the text to read (**lines 249-254**): "… Flow cytometry analyses confirmed a failure of $CD43^+$ blood cells to increase in *RUNX1*-KO cultures (19.9±3.1%, SOX- RUNX; 2.9±0.3%, RUNX1-KO; n=4, P<0.01, Student's t-test) at d2 of methylcellulose culture (Fig. 4F). By d5 (Fig. 4G and Fig. S5E) and d8 (Fig. S5D, E) of methylcellulose culture, more striking reductions in $CD43^+$, $GYPA^+$ and $RUNX1C^+$ cells were noted in *RUNX1*-KO compared to SOX- RUNX cultures.

-Figure 4F: It is not clear these are erythroid colonies as stated in the text.

The statement made in the text (line 284-287) was: "… However, in *RUNX1*-KO cultures, the only clonogenic cells detected were at d2, when a small number of erythroid colonies were observed arising from vascular cores when cells were cultured at high density in methylcellulose (Fig. 5F, G)." **Response**: Figure 5F shows a bar graph indicating the small numbers of colonies observed arising from high density cultures of *RUNX1*-KO cells, and the erythroid nature of these is shown

© 2020. Published by The Company of Biologists under the terms of the Creative Commons Attribution License (https://creativecommons.org/licenses/by/4.0/).

in Figure 5G.

-I may have missed this, but did not see a list of Taqman probe sets.
Response: We have corrected this oversight and the list of Taqman probe sets have now been included in the methods.

Second decision letter

MS ID#: DEVELOP/2020/193037

MS TITLE: Human yolk sac-like haematopoiesis generates RUNX1- and GFI1/1B-dependent blood and SOX17-positive endothelium

AUTHORS: Freya F Bruveris, Elizabeth S Ng, Ana Rita Leuitoguinho, Ali Motazedian, Katerina Vlahos, Koula Sourris, Robyn Mayberry, Penelope McDonald, Lisa Azzola, Nadia M Davidson, Alicia Oshlack, Edouard G Stanley, and Andrew G Elefanty
ARTICLE TYPE: Research Article

I am happy to tell you that your manuscript has been accepted for publication in Development, pending our standard ethics checks.

Reviewer 1

Advance summary and potential significance to field

The authors have adequately addressed the minor concerns raised, with modifications to the text. I have no other concerns.

Comments for the author

No further revisions are required/requested.

Reviewer 2

Advance summary and potential significance to field

In this manuscript entitled "Human yolk sac-like hematopoiesis generates RUNX1- and GFI1/1B-dependent blood and SOX17-positive endothelium," the authors explore the genetic regulatory network controlling early human blood development. Their main conclusion is that human blood cell development arises from a hemangioblast-like SOX17- endothelial cell population, and is sequentially dependent upon GFI1/1B and RUNX1.

The manuscript is well organized, and the results very interesting and important. The experiments presented are well performed, conclusive, and the interpretations sound. Although the findings presented are not entirely unexpected, given the results previously obtained in the mouse, they are definitely worth publication as it was never shown in the human setting.

Comments for the author

The authors have appropriately addressed my concerns. I have only a few minor remaining comments:

- Line 232: should it not be ITGA2B for the gene instead of ITA2B. My understanding is that ITA2B (or CD41) refers to the protein. Similar remark for ITGB3.

© 2020. Published by The Company of Biologists under the terms of the Creative Commons Attribution License (https://creativecommons.org/licenses/by/4.0/).

- In the new data presented upon replating of SOX17neg cells (movie and figure 2), most of the round cells generated do not express RUNX1C. Are these cells representing primitive erythroid cells? Is the generation of these cells blocked with LSD1i?

Reviewer 3

Advance summary and potential significance to field

The authors have answered most of my concerns. The addition of the life imaging has strengthened their data and the revised manuscript is much improved.

Comments for the author

I have no further comments.

© 2020. Published by The Company of Biologists under the terms of the Creative Commons Attribution License (https://creativecommons.org/licenses/by/4.0/).
